# Use of Antibiotics against Bacterial Infections on Dairy Sheep and Goat Farms: Patterns of Usage and Associations with Health Management and Human Resources

**DOI:** 10.3390/antibiotics11060753

**Published:** 2022-05-31

**Authors:** Daphne T. Lianou, George C. Fthenakis

**Affiliations:** Veterinary Faculty, University of Thessaly, 43100 Karditsa, Greece; dlianou@vet.uth.gr

**Keywords:** abortion, antibiotic, farmer, goat, health management, mastitis, neonatal diarrhoea, pneumonia, sheep, treatment

## Abstract

The objectives of the study were (a) to describe the patterns of antibiotic usage against four major clinical problems and (b) to evaluate factors that were associated with their use on small ruminant farms. Sheep and goat farmers mostly administered the antibiotics to animals at the dose prescribed (80.4%) and observed the necessary withdrawal period (98.7%), but fewer farmers (22.3%) weighed the animals to calculate their bodyweight before antibiotic administration. For the treatment of clinical mastitis, oxytetracycline, penicillin and streptomycin were the antibiotics used more frequently; 2.03 different antibiotics were used per sheep flock and 2.06 per goat herd, most frequently administered in injectable forms (88.8% of farms). In cases of abortion, oxytetracycline was administered more frequently; 1.12 different antibiotics were used per sheep flock and 1.03 per goat herd. In 94 farms (21.2%), routine administration of antibiotics was performed to newborns; oxytetracycline and ampicillin were administered more often. For the treatment of pneumonia in newborns, oxytetracycline, penicillin and tulathromycin were used more frequently; 1.33 antibiotics were used per sheep flock and 1.29 per goat herd. For the treatment of diarrhoea in lambs and kids, oxytetracycline, amoxicillin and penicillin were the antibiotics used more frequently; 1.34 antibiotics were used per sheep flock and 1.59 per goat herd. Results of multivariable analyses indicated 16 variables associated with the various outcomes for usage of antibiotics for the treatment of the above clinical problems. Of these, 11 variables were associated with the farmers: education of farmers was significant for three outcomes; the age, the experience, the professional involvement and farming family tradition of farmers and the daily period spent at the farm were each significant for one outcome.

## 1. Introduction

The appropriate and correct use of pharmaceutical products in sheep and goat farming is important for ensuring correct health management and maintenance of animal welfare standards on the farms, as well as for public health with regard to drug residues in animal products. Thus, knowledge of antibiotic usage patterns in the livestock industry can support the animal health industry to ensure that drugs are being used prudently.

In general, there is a scarcity of relevant information internationally regarding the use of pharmaceutical products, including antibiotics, on sheep and goat farms. In a relevant study performed in Canada, Moon et al. [1] collected information from 49 sheep flocks and reported that penicillin and oxytetracycline were the two most frequently used antibiotics. Another study performed in the United Kingdom, in 26 sheep farms, focusing on the potential associations between antibiotic administration and development of antimicrobial resistance, reported that farmers engaged in preventative measures, but they deflected responsibility for reducing antibiotic resistance to veterinarians [2]. In goat farms, Landfried et al. [3] investigated the patterns of antibiotic use in 11 goat herds and found that in only four farms were antibiotics administered based on a veterinary prescription.

Dairy sheep and goat farming is an important sector of the agricultural industry in Greece, with significant annual milk production. Data derived from the Hellenic Milk Board indicate that total deliveries of sheep and goat milk from these farms to dairy factories were approximately, respectively, 645,000 and 143,000 m^3^ [4]. These quantities amount to approximately 15% of the total European milk production from small ruminants [5] and confirm Greece as a significant producer of milk of small ruminants in Europe. Of those quantities, 90% was used for cheese production.

Despite the importance of small ruminant farming for the food production sector in Greece, the patterns of usage of antibiotics on the farms in cases of infections have not been investigated and described. However, there is scope for monitoring their usage, as recent investigations have highlighted the presence of bacterial resistance to antibiotics [6,7]. Moreover, potential associations of antibiotic administration with farm-related factors have not been reported.

This paper reports findings regarding antibiotic usage on small ruminant farms, as found during an extensive countrywide investigation performed in 325 sheep flocks and 119 goat herds throughout Greece. The objectives of the study were (a) to describe the patterns of antibiotic usage against four major infections on small ruminant farms and (b) to highlight factors that were associated with their use on the farms.

## 2. Materials and Methods

### 2.1. Small Ruminant Farms and Collection of Information

**Figure 1:**
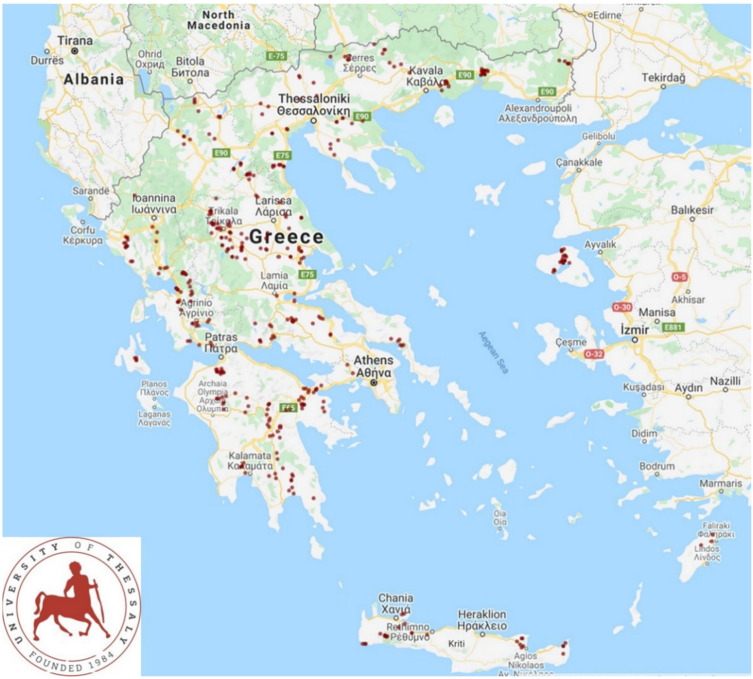
Location of the 444 small ruminant farms around Greece, which were visited to record details on the use of antibiotics.

A cross-sectional study involving 325 sheep flocks and 119 goat herds was performed from April 2019 to July 2020. The work covered all the 13 administrative regions of Greece (Figure 1). The flocks and herds were included in the study on a convenience basis (willingness of shepherds and goatherds to accept a visit by university personnel for interview and sample collection), as detailed previously [8]. The investigators visited all the flocks and herds for sample collection.

Veterinarians active in small ruminant health management around Greece were contacted by telephone and asked if they wished to collaborate in the investigation [8]; 47 veterinarians agreed to that request.

Initially, the veterinarian accompanying the investigators to the respective farm introduced them to the farmer. The senior investigator (author G.C.F.) explained to the farmer the objectives and the details of the study and also introduced the interviewer (author D.T.L.) to the farmer. 

The interview was performed using a structured detailed questionnaire. Before the start of the main study, the questionnaire used to collect data from the farmers had been tested for content validity [8]. The interview included general questions, as well as questions regarding infrastructure, animals, production characteristics, health management and human resources on the farm [8]. If farmers asked for clarifications of the questions during the interview, these were provided immediately by the interviewer. The mean (±standard error of the mean) duration of the interview was 63.6 ± 0.3 min [8]. After completing the interview, no repeat visits were made to the farms.

### 2.2. Samplings and Laboratory Examinations

During the visit to each sheep flock and goat herd, four milk samples (20 mL each) were collected aseptically directly from the bulk-tank of the farm. Two milk samples were used for composition analysis and somatic cell counting and the other two were used for bacteriological examinations.

Before laboratory analysis, two 10 mL subsamples were created and processed; therefore, each separate test was performed four times (each one in different subsamples). Milk composition and somatic cell counting were performed within 4 h after collection on each of the four relevant subsamples, as previously detailed [9,10,11]. Moreover, milk samples were processed for total bacterial counts, and culturing was performed for isolation and identification of staphylococcal species as described previously [9,10,11].

Transportation of the samples to the laboratory was made by the investigators and by car; samples collected from farms in the islands were also transported as accompanying luggage by airplane (Crete, Lesvos, and Rhodes) or by boat (Cephalonia). Samples were transported in portable refrigerators at 0.0 to 4.0 °C.

### 2.3. Data Management and Analysis

Data were entered into Microsoft Excel and analysed using SPSS v. 21 (IBM Analytics, Armonk, NY, USA). Details of the following general characteristics of usage of antibiotics on the farms were assessed: method of calculation of bodyweight for the administration of antibiotics, administration of antibiotics to animals at the dose prescribed, observation of the withdrawal period after administration of antibiotics and routine administration of antibiotics to newborn lambs/kids. Moreover, the following four clinical problems were studied in detail: mastitis and abortion in ewes and does and pneumonia and diarrhoea in lambs and kids; these four clinical entities are significant problems and important causes of production losses in small ruminant farms and their therapeutic management includes the administration of antibiotics. Initially, basic descriptive analyses were performed. Exact binomial confidence intervals (CI) were obtained.

The analysis results for the two subsamples from each of the two milk samples collected from the bulk tank were averaged, then the two means were again averaged for the final result regarding each bulk-tank milk. For statistical analysis, the somatic cell counts were transformed to somatic cell scores and the total bacterial counts were transformed to log_10_ as described previously [9,10,11]; the transformed data were used in the analyses. Differences were determined by using analysis of variance.

The following eight outcomes were considered: ‘*calculation of bodyweight for the administration of antibiotics by weighing*’, ‘*administration of antibiotics to animals at the dose prescribed*’, ‘*observation of the withdrawal period after administration of antibiotics*’, ‘*the number of antibiotics used for the treatment of clinical mastitis*’, ‘*use of antibiotics in cases of abortion*’, ‘*routine administration of antibiotics to newborn lambs/kids*’, ‘*use of antibiotics for the treatment of pneumonia in lambs/kids*’, ‘*use of antibiotics for the treatment of diarrhoea in lambs/kids*’. Τhe variables evaluated for potential associations with the above outcomes are in Table S1; these variables were either taken directly from the answers of the interview performed at the start of the visit or they were calculated based on these answers (e.g., total milk quantity per female animal obtained during the preceding milking period, which was calculated from the annual amount of milk produced and the number of females on the farm). For each of these variables, categories were created according to the answers of the farmers. Separate analyses were performed for sheep flocks and goat herds. Exact binomial CIs were obtained. The importance of predictors was assessed by cross-tabulation with Pearson’s chi-squared test and with simple logistic regression or by analysis of variance as appropriate. Separate univariable analyses were performed for each of the above eight outcomes. Subsequently, for the evaluation of each outcome, a different multivariable model was created, initially offering to the model all variables, which achieved a significance of *p* < 0.2 in the respective univariable analyses. Variables were removed from the initial model by backward elimination. The *p*-value of removal of a variable was assessed by the likelihood ratio test, and for those with a *p*-value of >0.2, the variable with the largest probability was removed. This process was repeated until no variable could be removed with a value of >0.2. The variables required for the final multivariable models constructed for each outcome are shown in Table S2.

In all analyses, statistical significance was defined at *p* < 0.05.

## 3. Results

### 3.1. General Characteristics of Usage of Antibiotics on the Farms

#### 3.1.1. Descriptive Results

The majority of farmers reported that they administered the antibiotics to animals at the dose prescribed (*n* = 357; 80.4% (76.5–83.8%)) and that they observed the withdrawal period after administration of antibiotics (*n* = 438; 98.7% (97.1–99.4%)). Fewer farmers responded that they weighed the animals for calculation of the proper dosage for the administration of antibiotics (*n* = 99; 22.3% (18.7–26.4%)). In total, 74 farmers (16.7% (13.5–20.4%)) performed all three practices correctly (Table 1).

#### 3.1.2. Associations with Variables Studied

**Figure 2:**
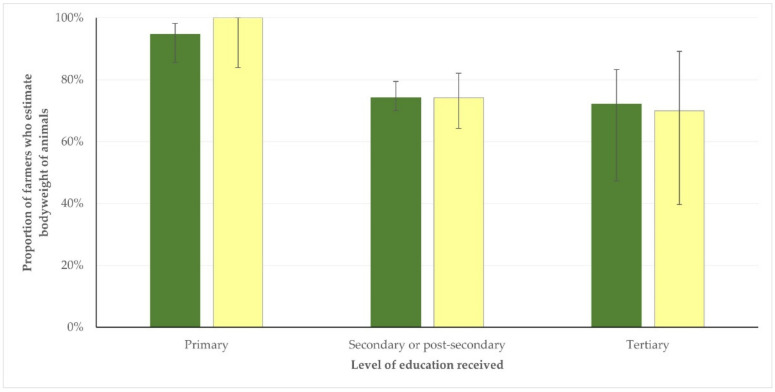
Education level of sheep (green) and goat (yellow) farmers in Greece, who calculated bodyweight for the administration of antibiotics by estimation (bars indicate 95% confidence intervals). (Correspondence of Education levels with the European Qualifications Framework Levels 1–8 are described in Table S1.)

The results of the univariable analyses for assessment of potential associations of these practices with the studied variables are in Tables S3–S5. During the multivariable analysis, for the method of calculation of bodyweight for the administration of antibiotics, significant associations were found with the education of the farmer (*p* = 0.007) and the number of animals on the farm (*p* = 0.020) for sheep farms and with the education of the farmer (*p* = 0.018) for goat farms (Figure 2). For the administration of antibiotics to animals at the dose prescribed, a significant association was found for sheep farms only, specifically with the number of animals on the farm (*p* = 0.029); for goat farms, no significant associations emerged (*p* > 0.37 for all comparisons). For the observation of the withdrawal period, a significant association was found for sheep farms only, specifically with the farmer’s education (*p* = 0.030); for goat farms, only a tendency emerged with the farmer’s involvement in the work (*p* = 0.08). The results of the multivariable analyses are in Table 2 and Table S2.

### 3.2. Use of Antibiotics in Cases of Clinical Mastitis

#### 3.2.1. Antibiotics Used

Of the 325 sheep flocks and 119 goat herds in the study, cases of clinical mastitis were reported in 272 (83.7%, 95% CI: 79.3–87.3%) and 71 (59.7%, 95% CI: 50.7–68.0%), respectively (*p* < 0.0001). The annual incidence rates of clinical mastitis in sheep flocks and goat herds were 3.9% (95% CI: 3.8–4.0%) and 2.8% (95% CI: 2.6–3.0%), respectively (*p* < 0.0001). Of these, treatment of the cases of mastitis was performed in 270 flocks (99.3%) and 71 herds (100.0%) (*p* = 0.47).

On 302 farms (88.6%) (236 flocks and 66 herds), treatment of clinical mastitis involved only the administration of antibiotics. On the remaining 39 farms (11.4%) (34 flocks and 5 herds) (*p* = 0.19), treatment involved the simultaneous administration of antibiotics and the non-steroid anti-inflammatory agent flunixin meglumine, which can be administered to alleviate pain, inflammation and fever in affected animals.

**Figure 3:**
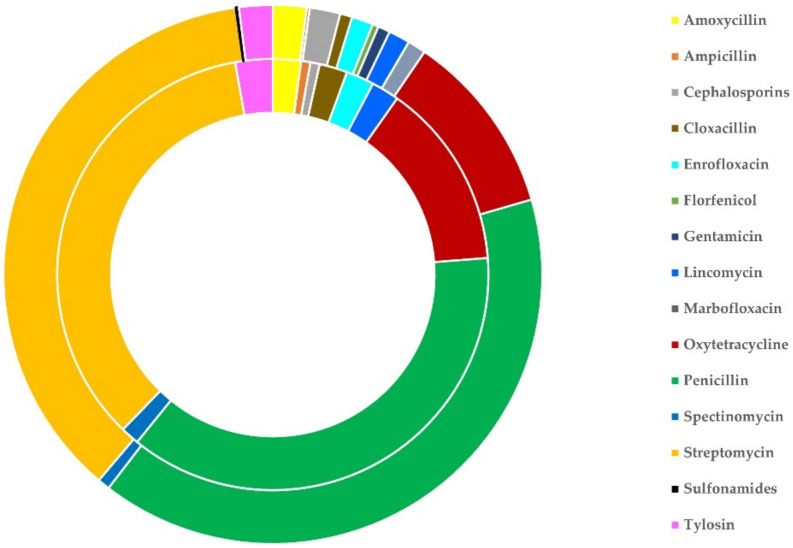
Schematic illustration of the sheep flocks (outer circle) and goat herds (inner circle) on which various antibiotics were administered in cases of clinical mastitis in Greece.

On average, for the treatment of clinical mastitis, 2.03 ± 0.04 antibiotics were used per sheep flock and 2.06 ± 0.09 antibiotics were used per goat herd (*p* = 0.81). The most frequently used antibiotic on its own was oxytetracycline (in 25 flocks, 9.3%, and 7 herds, 9.9%), whilst the most frequently used combination of antibiotics was penicillin with streptomycin (in 151 flocks, 55.9%, and 30 herds, 42.3%). These three antibiotics (on their own or in various combinations) were used in 219 flocks (81.1%) and 64 herds (90.1%). The frequency of use of the various antibiotics used is detailed in Table 3 and illustrated in Figure 3. There was no difference in the antibiotics used among farms with different management systems or farms with application of machine- or hand-milking for sheep and goats (*p* > 0.18 for all comparisons) (Table S6).

#### 3.2.2. Pharmaceutical Forms Used for Antibiotic Administration

On both sheep and goat farms, administration of antibiotics was most frequently performed in injectable form only (240 flocks, 88.9%, and 63 herds, 88.7%) for intramuscular or subcutaneous injection according to the licenced route of administration. Less frequently, it was performed in intramammary form only (19 flocks, 7.0%, and 8 herds, 11.3%), whilst, in some sheep flocks, both forms were employed (11 flocks, 4.1%) (*p* = 0.12).

There was evidence on both sheep and goat farms that the use of an injectable form was associated with a higher number of antibiotics being used for the treatment of clinical mastitis on that farm (*p* < 0.0001 in sheep flocks, *p* = 0.026 in goat herds) (Table 4). Moreover, there was also a tendency that in flocks where administration of antibiotics was performed in intramammary form, somatic cell counts and total bacterial counts of the bulk tank milk were lower: 0.393 × 10^6^ versus 0.501 × 10^6^ and 0.743 × 10^6^ cells mL^−1^ (*p* = 0.07) and 309 × 10^3^ versus 407 × 10^3^ and 1413 × 10^3^ cfu mL^−1^ (*p* = 0.035), respectively (Table S7).

#### 3.2.3. Factors Associated with the Number of Antibiotics Used in Cases of Clinical Mastitis

There was an association between higher incidence rate of clinical mastitis in sheep flocks and administration of antibiotics for its treatment: antibiotics were administered in 94.1% of flocks with incidence rate up to 1.0% and in 100% of flocks with incidence rate over 1.0% (*p* = 0.0002) (Table 5). However, there was no evidence that the number of antibiotics used was associated with the milk production and quality parameters (Table S7).

**Figure 4:**
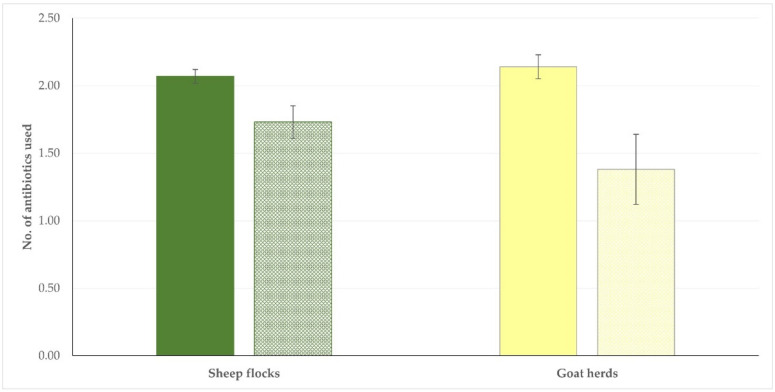
Numbers of antibiotics used on sheep (green) and goat (yellow) farms for the treatment of clinical mastitis in accordance with the professional involvement of farmers (full pattern: full-time farmers, motif pattern: part-time farmers) in Greece.

The results of the univariable analyses for assessment of potential associations of farm-related factors with the number of antibiotics used on the farms are in Tables S8 (sheep flocks) and S9 (goat herds). During the multivariable analysis, for sheep and goat farms, only the farmers’ professional involvement in the work emerged as a significant factor (*p* = 0.011 for sheep flocks, *p* = 0.015 for goat herds) (Table 6 and Table S2; Figure 4).

### 3.3. Use of Antibiotics in Cases of Abortion

#### 3.3.1. Antibiotics Used

Of the 325 sheep flocks and 119 goat herds in the study, cases of abortion were reported in 154 (47.4%, 95% CI: 42.0–52.8%) and 57 (47.9%, 95% CI: 39.1–56.8%), respectively (*p* = 0.92). The annual incidence rates of abortion in sheep flocks and goat herds were 2.0% (95% CI: 1.9–2.1%) and 2.7% (95% CI: 2.5–2.9%), respectively (*p* < 0.0001). Of these, use of antibiotics was reported in 68 flocks (44.2%) and 40 herds (70.2%) (*p* = 0.0008).

On all the farms with cases of abortion (100.0%), use of pharmaceutical products involved only the administration of antibiotics.

**Figure 5:**
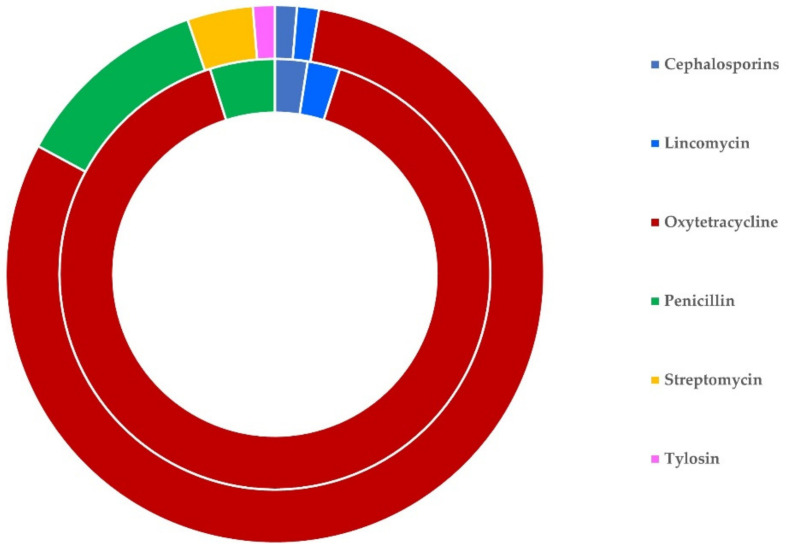
Schematic illustration of the sheep flocks (outer circle) and goat herds (inner circle) in which various antibiotics were administered in cases of abortion, as found in a countrywide investigation in Greece.

On average, 1.12 ± 0.05 antibiotics were used per flock and 1.03 ± 0.03 antibiotics were used per herd (*p* = 0.17). The most frequently used antibiotic was oxytetracycline (in 61 flocks, 89.7%, 37 herds, 92.5%). The frequency of the various antibiotics used is detailed in Table 7 and illustrated in Figure 5.

#### 3.3.2. Factors Associated with the Administration of Antibiotics in Cases of Abortion

There was no association between the incidence rate of abortion on the farms and the administration of antibiotics (*p* = 0.44 for sheep flocks, *p* = 0.21 for goat herds; Table S10).

The results of the univariable analyses for assessment of potential associations of farm-related factors with the use of antibiotics on the farms are in Tables S11 (sheep flocks) and S12 (goat herds). During the multivariable analysis, for sheep and goat farms, farmer-related characteristics emerged primarily as significant factors. Specifically, for sheep farms, the experience of the farmer (*p* = 0.001), the daily period spent at the farm (*p* = 0.010) and the age of the farmer (*p* = 0.012), and additionally the season of the start of the lambing period (*p* = 0.007) were found as significant factors (Table 8 and Table S2). For goat farms, the age of the farmer (*p* = 0.05) emerged as a significant factor (Table 8 and Table S2).

### 3.4. Routine Administration of Antibiotics to Newborn Lambs/Kids

Of the 325 sheep flocks and 119 goat herds in the study, routine administration of antibiotics to newborn lambs/kids was reported in 65 (20.0%, 95% CI: 16.0–24.7%) and 29 (24.4%, 95% CI: 17.5–32.8%), respectively (*p* = 0.32).

**Figure 6:**
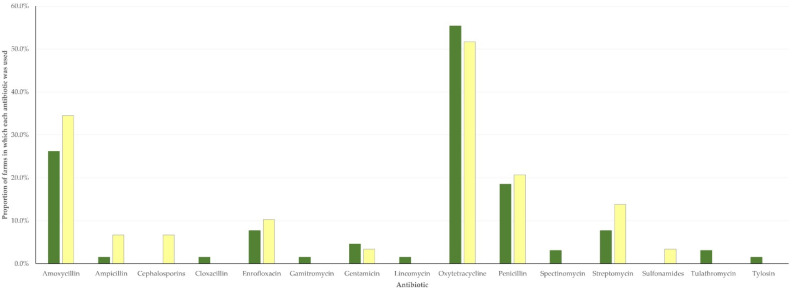
Proportion of small ruminant farms (n), on which various antibiotics were used during routine administration to newborn lambs (green)/kids (yellow) in Greece.

The most frequently used antibiotic (on its own or in combination) was oxytetracycline (in 36 flocks, 55.4%, and 15 herds, 51.7%). Two antibiotics, namely oxytetracycline and amoxicillin (on their own or in various combinations) were the ones which were more frequently routinely administered to newborn lambs/kids; specifically, they were administered in 45 flocks (69.2%) and 20 herds (69.0%). The frequency of the various antibiotics used is detailed in Table 9 and illustrated in Figure 6.

The results of the univariable analyses for assessment of potential associations of this practice with the studied variables are in Table S13. During the multivariable analysis, for sheep farms, only the education of farmers (*p* = 0.047) emerged as a significant factor, whilst for goat farms no significant associations were found (*p* > 0.11) (Table 10 and Table S2).

**Figure 7:**
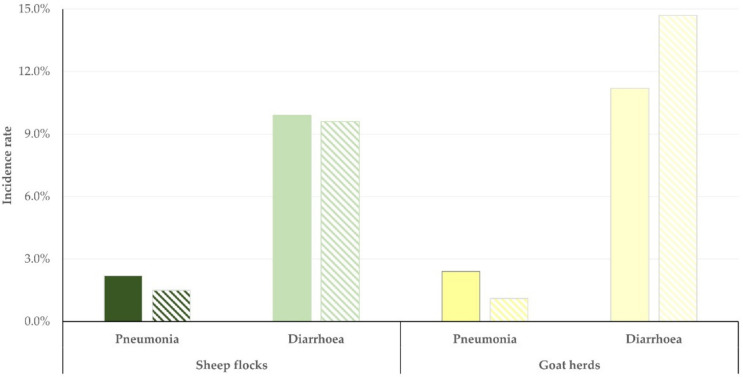
Incidence rate of pneumonia (dark shaded) or diarrhoea (light shaded) in lambs (green) or kids (yellow), in accordance with routine administration (motif pattern) or no administration (full pattern) of antibiotics in Greece.

The incidence rate of pneumonia in flocks and herds, in which antibiotics were routinely administered to newborns was clearly higher than on farms on which no such antibiotics were given (*p* < 0.0001). In contrast, the respective findings for the incidence rate of diarrhoea were conflicting (Figure 7, Table S14).

### 3.5. Use of Antibiotics in Cases of Pneumonia in Lambs/Kids

#### 3.5.1. Antibiotics Used

Of the 325 sheep flocks and 119 goat herds in the study, cases of pneumonia in lambs/kids were reported in 96 (29.5%, 95% CI: 24.8–34.7%) and 23 (19.3%, 95% CI: 13.2–27.3%), respectively (*p* = 0.031). The annual incidence rates of lamb/kid pneumonia in sheep flocks and goat herds were 1.7% (95% CI: 1.6–1.8%) and 1.5% (95% CI: 1.3–1.6%), respectively (*p* = 0.013). Of these, treatment of the cases of the disorder was performed in 71 flocks (74.0%) and 17 herds (73.9%) (*p* = 0.99).

On all the farms with cases of pneumonia in lambs and kids (100.0%), its treatment involved only the administration of antibiotics. In no case was the administration of non-steroid anti-inflammatory drugs reported.

**Figure 8:**
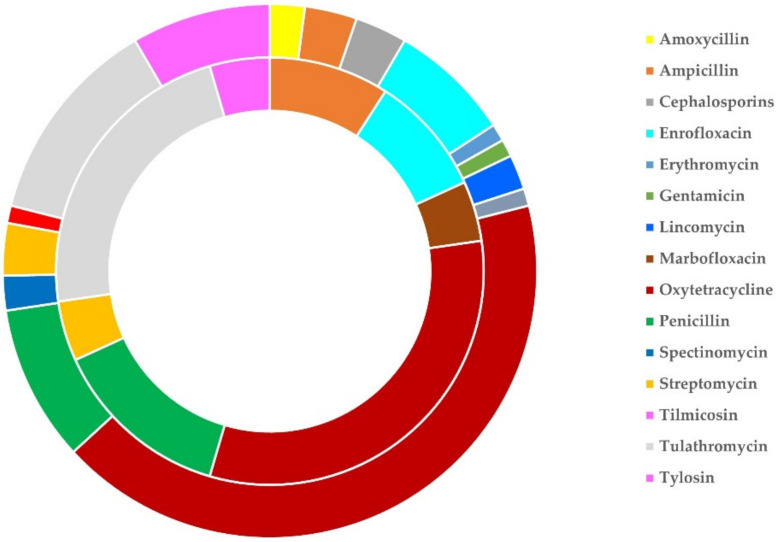
Schematic illustration of the sheep flocks (outer circle) and goat herds (inner circle) in which various antibiotics were administered in cases of pneumonia in lambs/kids, as found in a countrywide investigation in Greece.

On average, 1.33 ± 0.07 antibiotics were used per flock and 1.29 ± 0.11 antibiotics were used per herd (*p* = 0.83). The most frequently used antibiotic (on its own or in combination) was oxytetracycline (in 40 flocks, 56.4%, and 7 herds, 41.2%), whilst the most frequently used combination of antibiotics was oxytetracycline with penicillin (in four flocks, 5.6%, and one herd, 5.9%). Three antibiotics, namely oxytetracycline, penicillin and tulathromycin (on their own or in various combinations) were used in 56 flocks (78.9%) and 14 herds (58.3%). The frequency of the various antibiotics used is detailed in Table 11 and illustrated in Figure 8.

There was a clear difference between the frequency of antibiotics given routinely to lambs/kids and that of antibiotics given in cases of pneumonia (*p* = 0.003 for sheep farms, *p* = 0.023 for goat farms).

#### 3.5.2. Factors Associated with the Administration of Antibiotics in Cases of Pneumonia in Lambs/Kids

There was no association between the incidence rate of lamb or kid pneumonia on the farms and the administration of antibiotics (*p* = 0.21 for sheep flocks, *p* = 0.42 for goat herds; Table S15).

The results of the univariable analyses for assessment of potential associations of farm-related factors with the use of antibiotics on the farms are in Tables S16 (sheep flocks) and S17 (goat herds). During the multivariable analysis, for sheep and goat farms, farmer-related characteristics emerged again primarily as important factors. Specifically, for sheep farms, the family farming tradition was found to be significant (*p* = 0.043), whilst for goat farms, no significant associations emerged (*p* > 0.15) (Table 12 and Table S2).

### 3.6. Use of Antibiotics in Cases of Diarrhoea in Lambs/Kids

#### 3.6.1. Antibiotics Used

Of the 325 sheep flocks and 119 goat herds in the study, cases of diarrhoea in lambs/kids were reported in 194 (59.7%, 95% CI: 54.3–64.9%) and 69 (58.0%, 95% CI: 49.0–66.5%), respectively (*p* = 0.75). The annual incidence rates of lamb/kid diarrhoea in sheep flocks and goat herds were 9.6% (95% CI: 9.5–9.8%) and 13.7% (95% CI: 13.3–14.1%), respectively (*p* < 0.0001). Of these, treatment of the cases of the disorder was performed in 169 flocks (87.1%) and 64 herds (92.8%) (*p* = 0.21).

On 195 farms (141 flocks and 54 herds), treatment of diarrhoea in lambs and kids involved the administration of antibiotics. On 62 farms (41 flocks and 21 herds) treatment involved solely (28 and 10, respectively) or in addition to antibiotics (13 and 11, respectively), the administration of anticoccidial drugs (diclazuril, halofuginone, paromomycin, toltrazuril) in the therapeutic schemes.

**Figure 9:**
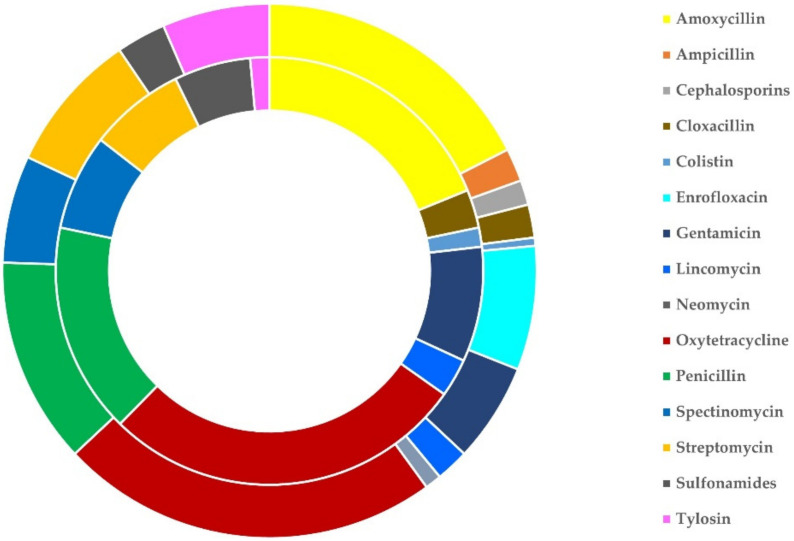
Schematic illustration of the sheep flocks (outer circle) and goat herds (inner circle) in which various antibiotics were administered in cases of diarrhoea in lambs/kids, as found in a countrywide investigation in Greece.

On average, 1.34 ± 0.05 antibiotics were used per flock and 1.59 ± 0.30 antibiotics were used per herd (*p* = 0.21). The most frequently used antibiotic (on its own or in combination) was oxytetracycline (in 46 flocks, 27.2%, and 19 herds, 35.2%), whilst the most frequently used combination of antibiotics was penicillin with streptomycin (in 16 flocks, 11.2%, and 4 herds, 7.4%). Three antibiotics, namely oxytetracycline, amoxicillin and penicillin (on their own or in various combinations) were used in 103 flocks (60.9%) and 38 herds (70.4%). The frequency of the various antibiotics used is detailed in Table 13 and illustrated in Figure 9.

There was some difference between the antibiotics given routinely to lambs/kids and to antibiotics given in cases of diarrhoea (*p* = 0.040 for sheep farms, *p* = 0.07 for goat farms).

#### 3.6.2. Factors Associated with the Administration of Antibiotics in Cases of Diarrhoea in Lambs/Kids

There was no association between the incidence rate of lamb or kid diarrhoea on the farms and the administration of antibiotics (*p* = 0.45 for sheep flocks, *p* = 0.35 for goat herds; Table S18).

The results of the univariable analyses for assessment of potential associations of farm-related factors with the use of antibiotics on the farms are in Tables S19 (sheep flocks) and S20 (goat herds). During the multivariable analysis, for sheep farms, the administration of antibiotics to animals at the dose prescribed (*p* = 0.017) and the routine administration of antibiotics to newborns (*p* = 0.048) emerged as significant factors, whilst for goat farms, no significant results were found (*p* > 0.10) (Table 14 and Table S2).

### 3.7. Summary of Results of Multivariable Analyses

Results of the multivariable analyses with a statistical significance are summarized in Table 15. Overall, significance was found in sheep flocks for the eight outcomes assessed and in goat herds for three of them. Of the 16 variables that were found to have a significant association with the various outcomes (13 in sheep flocks and 3 in goat herds), 11 were associated with the farmers (8 in sheep flocks and 3 in goat herds). Specifically, education of farmers was significant for three outcomes; the age, the experience, the professional involvement and farming family tradition of farmers, as well the daily period spent at the farm were each significant for one outcome.

## 4. Discussion

### 4.1. Preamble

Small ruminant farming is currently the most important animal farming business in Greece, generating 18% of the total primary sector income [12]. Small ruminant farming in Greece is characterized primarily by dairy production. In this system, lambs and kids are weaned at the age of 5 to 90 days and then are sent for slaughter at the age of 40 to 120 days. Ewes and does are thereafter milked for an additional 3 to 8 months; milk is sold to dairy factories for the preparation of cheese or yoghurt. The importance of small ruminant farming for Greek agriculture is illustrated by the fact that national annual milk production from small ruminants exceeds that from cattle [13].

The present study investigated patterns of antibiotic usage in small ruminant farms in an extensive countrywide investigation of 444 farms. Moreover, the study evaluated potential associations with some management practices and the human resources on the farms. Farms from all regions of Greece were included in the study, allowing that situations and conditions present in all the parts of the country were taken into account and factors of regional importance weighed less. As far as we are aware, and to the best of our knowledge, this was the largest sample size used to investigate these issues. The farms studied in the present work represent approximately 1% of the total sheep and goat farms in the country, in accordance with the data of the Hellenic Milk Board [4]. Although farms were enrolled in the study on a convenience basis, this approach guaranteed acceptance by the farmers of the visit and lack of suspiciousness and distrust of the investigators, resulting in a relaxed interview. In order to minimize possible bias, the study used consistent methodologies and ensured that the interviews were always performed by the same investigator.

Inappropriate antibiotic use in food animals can contribute to development of resistance to antibiotics, and relevant evidence has been available for many years [14]. In turn, this may represent a potential risk to human health [14]. In farm animals, antibiotics are used to treat clinical diseases, as well as to control common infections (e.g., prophylactic administration in female animals after a case of dystocia, in which case there is increased risk for development of post-partum endometritis). However, there is a paucity of data internationally regarding the patterns of usage of antibiotics, despite their importance, as discussed hereabove [15].

Convenience selection of study farms was employed, which allowed inclusion of flocks and herds with farmers willing to participate in the study and provide relevant answers. Some degree of stratification was also applied, as farms from all 13 administrative regions of the country were included in the study. The established limitations of questionnaire surveys certainly also applied in this work (e.g., differences in understanding and interpretation, inclusion of respondents with a personal agenda, unconscientious responses) [16]. However, efforts were made to minimize any possible adverse effects in the study; for example, any queries of the interviewees were answered immediately by the interviewer (author D.T.L.) [8], whilst the second author (G.C.F.) discussed some of the farmers’ answers with the veterinarians accompanying in the farms, with the aim to verify them [8].

### 4.2. General Characteristics of Usage of Antibiotics on the Farms

The majority of farmers observed the withdrawal period after antibiotic administration and also administered the drugs at the dose prescribed. However, only few farmers weighed the animals for accurate calculation of dosage per bodyweight before drug administration. This practice certainly includes a contradiction as the estimation of the bodyweight of animals may not always lead to correct guessing of the actual bodyweight of animals. Visual estimation of the weight of animals can lead to errors, especially in crossbreed animals [17] as body structure can be deceptive for accurately calculating actual bodyweight [18], particularly in goats, which often have light bones [17,19].

Strict enforcement of regulations and the application of continuous assessments for detection of residues in animal products in the country motivate farmers to maintain withdrawal periods after antibiotic use. Nevertheless, a recent study in Greece reported that in 12.1% of raw milk samples from sheep, antibiotic residues were detected [20], which is higher than the proportion of farmers skipping withdrawal periods. Hence, it becomes evident that although the majority of farmers would observe withdrawal periods, residues may still be detected in milk, as the result of other mistakes during usage of antibiotic products, potentially highlighting the need for the determination of accurate bodyweight.

### 4.3. Use of Antibiotics for Infections in Ewes and Does

In cases of mastitis in small ruminants, early initiation of treatment is important, to minimize mammary lesions and to restore the health of the affected animals. Ideally and to preserve susceptibility of pathogens to the available drugs, treatment must be performed using a narrow-spectrum drug specifically effective against the causal bacteria of each particular case; administration of the drug should follow identification of the bacteria and establishment of their susceptibility patterns. Nevertheless, this may not be always possible, because of two conflicting factors: (i) the necessity for early instigation of treatment and (ii) the time required to perform a complete bacteriologic examination (including bacterial isolation, identification and susceptibility testing) [21]. Therefore, treatment may potentially start blindly by means of a broad-spectrum or a combination of narrow-spectrum antibiotics effective against the major causal agents of the disease. This practice became evident to some extent in the present findings, in which oxytetracycline, a broad-spectrum agent, or a combination of penicillin and streptomycin were the most frequently employed products. Thus, ‘first-line’ antibiotics were more often administered for the treatment of mastitis on the farms in the study.

A non-steroid anti-inflammatory agent, in all cases flunixin meglumine, was used in only 11% of farms. Nevertheless, the use of flunixin meglumine in cases of mastitis promotes rapid resolution of clinical signs [22,23], thus improving the welfare of affected animals [24], and should henceforth be advocated and applied as an adjunct treatment in small ruminant mastitis.

The increased proportion of farmers preferring the systematic administration of antibiotics in comparison to the intramammary route may possibly reflect the lack of antibiotic products specifically licenced for use in small ruminants. This use is clearly reflected in the significantly smaller number of antibiotics used for the treatment of the infection in farms using intramammary administration in comparison to farms using injectable or mixed administration. Whilst, in principle, treatment of mastitis should be performed by using intramammary antibiotic tubes [21], obviously the limited array of antibiotics available for small ruminants forces farmers to consider as main therapeutic options the injectable route.

Oxytetracycline is used in the vast majority of outbreaks of abortion, as the drug is administered metaphylactically in cases caused by *Chlamydia abortus* or *Coxiella burnetii* [25,26], which constitute two main causes of the problem in small ruminants, to prevent abortion by other pregnant animals in the flock or herd. Apart from the metaphylactic use, oxytetracycline is also given to animals that aborted, to prevent development of uterine infection post-abortion.

### 4.4. Administration of Antibiotics to Lambs/Kids

The rationale for routine administration of antibiotics to newborns is the prevention of neonatal infections in these animals. This practice should be discouraged, as, first and foremost, it is a paramount factor for the development of antibiotic resistance among bacteria prevailing on the farms. Moreover, from the viewpoint of the potential efficacy, the results do not justify the practice: the incidence rate of pneumonia was higher on farms, on which the practice was taking place and no beneficial effects were seen for the incidence rate of diarrhoea on the farms. In the first case, it should be noted that pneumonia in newborns is predisposed by various management-related factors [27]. Errors in health management in sheep and goat farms are often important factors precipitating the development of the infection [27] and must be corrected to control the infection on a farm. In fact, correction of such errors is more important than antibiotic administration in preventing the occurrence of pneumonia in newborns on a farm [28]. Thus, one may hypothesize that, on farms with high incidence rates of pneumonia in lambs or kids, farmers attempted to control the infection by preventive administration of antibiotics rather than targeting inappropriate management. In the second case, it should be noted that diarrhoea in lambs and kids is caused by a variety of causal agents, which, apart from bacteria, also include protozoa and viruses (e.g., *Cryptosporidium* spp., *Giardia* spp., *Rotavirus*) [29]. These are pathogens against which antibiotics are not efficient. Moreover, management factors (e.g., increased stocking rate in animal sheds) also play a role in the development of the problem. Hence, the above indicate that there may be little merit in the prophylactic administration of antibiotics against diarrhoea.

The above become particularly evident from the significant differences between the antibiotics given routinely to lambs/kids and those administered in cases of pneumonia or diarrhoea on the farms. This confirms the development of infection despite a prophylactic administration of antibiotics and the necessity for administering other products to treat the infections (e.g., tulathromycin for the treatment of pneumonia).

### 4.5. Factors Associated with the Administration of Antibiotics

The results of the univariable and multivariable analyses performed with regard to the use of antibiotics on the farms indicated that in most circumstances human resources were the predictors with significant associations for the use of antibiotics on the farms. In fact, human resource-related variables were found to be more important than factors related to management on the farms.

Complex interactions exist as part of various-type social contacts (e.g., between animals within a farm, between farmers and animals, between domestic animals and wildlife) as part of various procedures (e.g., various everyday farming activities (for example, milking or grazing), storage, food preparation) or between the physical environment (e.g., air, soil, water), as well as in human use patterns (e.g., food preparation, meat consumption, susceptibility to infection) [14].

With regard to the consumption of antibiotics by people, socio-demographic characteristics were found to be important. Among these traits, people who lived in farming regions were the ones with significantly higher antibiotic consumption [30]. Additionally, the same authors suggested that parents often influence children’s perceptions about antibiotic use [30]. In other studies, education level was also found as a determinant of consumption of antibiotics by healthy individuals or people with an infection [31].

The present findings indicate that similar trends are apparent in the usage of antibiotics for animals and socio-demographic characteristics of farmers are thus important determinants of the antibiotic usage patterns. Hence, education and updating of farmers regarding correct use of antibiotics should be continued, which can help achieve the prevention of the development of antibiotic resistance, as well as improved treatment of sheep and goat diseases on the farms.

Extensive campaigns on antibiotic stewardship have been performed aiming to encourage prudent use of antibiotics and to limit their unnecessary usage. The ultimate objective of such actions is to preserve their efficacy for serious and life-threatening infections of humans [32,33].

## 5. Conclusions

The study explored the antibiotic usage patterns on small ruminant farms. The study presented another facet of the interactions between people and farm animals in the food-producing chain. Improved antimicrobial usage, based on correct scientific principles and compliance with regulations and policies, coupled with surveillance on the farms are important for improving the welfare of farm animals and also minimizing development of antibiotic resistance. These people–animal interactions can possibly be considered as another approach within the ‘One Health’ concept. Through the present results, the importance of training farmers regarding correct usage of antibiotics became prominent. In turn, correct use of antibiotics, as guided by surveying studies, will contribute to a reduction in bacterial resistance to these drugs.

## Figures and Tables

**Table 1:** General characteristics of usage of antibiotics on small ruminant farms in Greece.

	Sheep Flocks (*n* = 325)	Goat Herds (*n* = 119)	*p*
Method of calculation of bodyweight for the administration of antibiotics			
Estimation	252 (77.5% (72.7–81.7%)) ^1^	93 (78.1% (68.9–84.6%))	0.96
Weighing	73 (22.5% % (18.3–27.3%))	26 (21.9% (15.4–30.1%))	
Administration of antibiotics to animals at the dose prescribed			
At the dose prescribed	264 (81.2% (76.6–85.1%))	93 (78.1% (68.9–84.6%))	0.47
At higher dose than prescribed	61 (18.8% (14.9–23.4%))	26 (21.9% (15.4–30.1%))	
Observation of the withdrawal period after administration of antibiotics			
Yes	321 (98.8% (96.9–99.5%))	117 (98.3% (94.1–99.5%))	0.72
No	4 (1.2% (0.5–3.1%))	2 (1.7% (0.5–5.9%))	

^1^ number of farms (proportion [95% confidence interval]).

**Table 2:** Multivariable analysis for variables associated with the general characteristics of usage of antibiotics on small ruminant farms in Greece.

Variables	Odds Ratio ^1^ (95% Confidence Intervals)	*p* Value
**Calculation of Bodyweight for the Administration of Antibiotics by Weighing**		
Sheep flocks		
Education of farmer		0.007
Primary (3/57 = 5.3% ^2^)	reference	-
Secondary or post-secondary (58/225 = 25.8%)	6.252 (1.882–20.764)	0.003
Tertiary (12/43 = 27.9%)	6.968 (1.824–26.611)	0.005
Number of animals on the farms		0.020
≤165 ewes (13/88 = 14.8%)	reference	-
166–330 ewes (26/120 = 21.7%)	1.596 (0.768–3.317)	0.21
331–500 ewes (20/56 = 35.7%)	3.205 (1.435–7.158)	0.005
>500 ewes (14/51 = 27.5%)	2.183 (0.932–5.115)	0.07
Goat herds		
Education of farmer		0.018
Primary (0/20 = 0.0%)	reference	-
Secondary or post-secondary (23/89 = 25.8%)	14.489 (0.843–249.133)	0.07
Tertiary (3/10 = 30.0%)	19.133 (0.880–415.906)	0.06
**Administration of Antibiotics to Animals at the Dose Prescribed**		
Sheep flocks		
Number of animals on the farms		0.029
≤165 ewes (79/88 = 89.8%)	2.701 (1.049–6.953)	0.039
166–330 ewes (96/120 = 80.0%)	1.231 (0.561–2.703)	0.60
331–500 ewes (50/56 = 89.3%)	2.564 (0.883–7.443)	0.08
>500 ewes (39/51 = 76.5%)	reference	-
**Observation of the Withdrawal Period**		
Sheep flocks		
Education of farmer		0.030
Primary (56/57 = 98.2%)	2.732 (0.240–31.156)	0.42
Secondary or post-secondary (224/225 = 99.6%)	10.927 (0.968–123.310)	0.05
Tertiary (41/43 = 95.3%)	reference	-

^1^ Odds ratios calculated against the lowest prevalence associations of the variable. ^2^ Numerator: no. of farms among those included in the denominator, in which the outcome of interest was seen; denominator: no. of farms in which the studied variable prevailed.

**Table 3:** Numbers of small ruminant farms (*n*) on which various antibiotics were administered for the treatment of clinical mastitis in Greece.

Antibiotic	Sheep Flocks (*n* = 270)	Goat Herds (*n* = 71)
Amoxicillin	11 (4.1%) ^1^	3 (4.2%)
Ampicillin	1 (0.4%)	1 (1.4%)
Cephalosporins	10 (3.7%)	1 (1.4%)
Cloxacillin	4 (1.5%)	3 (4.2%)
Enrofloxacin	7 (2.6%)	3 (4.2%)
Florfenicol	2 (0.7%)	0 (0.0%)
Gentamicin	4 (1.5%)	0 (0.0%)
Lincomycin	7 (2.6%)	3 (4.2%)
Marbofloxacin	6 (2.2%)	0 (0.0%)
Oxytetracycline	60 (22.2%)	20 (2.8%)
Penicillin	218 (80.7%)	53 (74.6%)
Spectinomycin	4 (1.5%)	2 (2.8%)
Streptomycin	200 (74.1%)	50 (70.4%)
Tylosin	11 (4.1%)	4 (5.6%)

^1^ number of farms (proportion).

**Table 4:** Numbers of antibiotics used on small ruminant farms for the treatment of clinical mastitis in accordance with the pharmaceutical form used on the farms for administration of antibiotics in Greece.

Pharmaceutical Form Used on the Farms for Administration of Antibiotics	Sheep Flocks	Goat Herds
Injectable forms	2.05 ± 0.04	2.13 ± 0.09
Forms for intramammary administration	1.42 ± 0.12	1.50 ± 0.19
Injectable forms and forms for intramammary administration	2.73 ± 0.24	*n*/a

**Table 5:** Association of the incidence rate of clinical mastitis with the administration of antibiotics on small ruminant farms in Greece.

Incidence Rate of Mastitis	Sheep Flocks		Goat Herds	
	Administration of Antibiotics in Cases of Mastitis	No Administration of Antibiotics in Cases of Mastitis	Administration of Antibiotics in Cases of Mastitis	No Administration of Antibiotics in Cases of Mastitis
≤1.0%	32 (94.1%) ^1^	2 (5.9%)	5 (100.0%)	0
>1.0%	238 (100.0%)	0 (0.0%)	66 (100.0%)	0
*p*	0.0002		n/a	

^1^ Number of sheep flocks/goat herds (proportion of total farms).

**Table 6:** Multivariable analysis for associations with the number of antibiotics used on farms for the treatment of clinical mastitis in Greece.

Variables	Odds Risk ^1^ (95% Confidence Intervals)	*p* Value
**Sheep Flocks**		
Professional involvement of farmer		0.011
Full-time farmers (2.07 ± 0.05 ^2^)	2.638 (1.488–11.608)	0.011
Part-time farmers (1.73 ± 0.12)	reference	-
**Goat Herds**		
Professional involvement of farmer		0.015
Full-time farmers (2.14 ± 0.09)	2.400 (1.776–7.136)	0.015
Part-time farmers (1.38 ± 0.26)	reference	-

^1^ Odds risks calculated against the lowest associations of the variable. ^2^ Mean ± standard error of the mean for the outcome of interest.

**Table 7:** Numbers of small ruminant farms (*n*) on which various antibiotics were used in cases of abortion in Greece.

Antibiotic	Sheep Flocks (*n* = 68)	Goat Herds (*n* = 40)
Cephalosporins	1 (1.5%) ^1^	1 (2.5%)
Lincomycin	1 (1.5%)	1 (2.5%)
Oxytetracycline	61 (89.7%)	37 (92.5%)
Penicillin	9 (13.2%)	2 (5.0%)
Streptomycin	3 (4.4%)	0 (0.0%)
Tylosin	1 (1.5%)	0 (0.0%)

^1^ Number of farms (proportion).

**Table 8:** Multivariable analysis for factors associated with the administration of antibiotics in cases in abortion in Greece.

Variables	Odds Ratio ^1^ (95% Confidence Intervals)	*p* Value
**Sheep Flocks**		
Experience of the farmer		0.001
≤5 years (12/41 = 29.3% ^2^)	reference	-
>5 years (56/113 = 49.6%)	2.374 (1.102–5.114)	0.027
Season of start of the lambing period		0.007
All year (3/9 = 33.3%)	1.500 (0.1056–21.313)	0.76
Autumn (44/75 = 58.7%)	4.258 (0.423–42.872)	0.22
Winter (20/66 = 30.3%)	1.304 (0.128–13.317)	0.82
Spring–Summer (1/4 = 25.0%)	reference	-
Daily period spent by the farmer at the farm		0.010
≤8 hours (13/46 = 28.3%)	reference	-
>8 hours (55/108 = 50.9%)	2.634 (1.251–5.546)	0.011
Age of farmer		0.012
≤50 years (51/98 = 52.0%)	2.489 (1.244–4.983)	0.010
>50 years (17/56= 30.4%)	reference	-
**Goat Herds**		
Age of farmer		0.05
≤50 years (29/37 = 78.4%)	3.296 (1.033–10.514)	0.44
>50 years (11/21 = 52.4%)	reference	-

^1^ Odds ratios calculated against the lowest prevalence associations of the variable. ^2^ Numerator: no. of farms among those included in the denominator, in which the outcome of interest was seen; denominator: no. of farms in which the studied variable prevailed.

**Table 9:** Numbers of small ruminant farms (*n*) on which various antibiotics were routinely administered to newborn lambs/kids in Greece.

Antibiotic	Sheep Flocks (*n* = 65)	Goat Herds (*n* = 29)
Amoxicillin	17 (26.2%) ^1^	10 (34.5%)
Ampicillin	1 (1.5%)	2 (6.7%)
Cephalosporins	0 (0.0%)	2 (6.7%)
Cloxacillin	1 (1.5%)	0 (0.0%)
Enrofloxacin	5 (7.7%)	3 (10.3%)
Gamitromycin	1 (1.5%)	0 (0.0%)
Gentamicin	3 (4.6%)	1 (3.4%)
Lincomycin	1 (1.5%)	0 (0.0%)
Oxytetracycline	36 (55.4%)	15 (51.7%)
Penicillin	12 (18.5%)	6 (20.7%)
Spectinomycin	2 (3.1%)	0 (0.0%)
Streptomycin	5 (7.7%)	4 (13.8%)
Sulfonamides	0 (0.0%)	1 (3.4%)
Tulathromycin	2 (3.1%)	0 (0.0%)
Tylosin	1 (1.5%)	0 (0.0%)

^1^ Number of farms (proportion).

**Table 10:** Multivariable analysis for factors associated with the routine administration of antibiotics to newborn lambs in Greece.

Variables	Odds Ratio ^1^ (95% Confidence Intervals)	*p* Value
Education of farmers		0.047
Primary (21/57 = 36.8% ^2^)	3.228 (1.710–6.278)	0.0003
Secondary or post-secondary (34/225 = 15.1%)	reference	-
Tertiary (10/43 = 23.3%)	1.702 (0.768–3.774)	0.19

^1^ Odds ratio calculated against the lowest prevalence associations of the variable. ^2^ Numerator: no. of farms among those included in the denominator, in which the outcome of interest was seen; denominator: no. of farms in which the studied variable prevailed.

**Table 11:** Numbers of small ruminant farms (*n*) on which various antibiotics were administered for the treatment of pneumonia in lambs or kids in Greece.

Antibiotic	Sheep Flocks (*n* = 71)	Goat Herds (*n* = 17)
Amoxicillin	2 (2.8%) ^1^	0 (0.0%)
Ampicillin	3 (4.2%)	2 (11.8%)
Cephalosporins	3 (4.2%)	0 (0.0%)
Enrofloxacin	7 (9.9%)	2 (11.8%)
Erythromycin	1 (1.4%)	0 (0.0%)
Gentamicin	1 (1.4%)	0 (0.0%)
Lincomycin	2 (2.8%)	0 (0.0%)
Marbofloxacin	1 (1.4%)	1 (5.9%)
Oxytetracycline	40 (56.3%)	7 (41.2%)
Penicillin	9 (12.7%)	3 (17.6%)
Spectinomycin	2 (2.8%)	0 (0.0%)
Streptomycin	3 (4.2%)	1 (5.9%)
Tilmicosin	1 (1.4%)	0 (0.0%)
Tulathromycin	12 (16.9%)	5 (29.4%)
Tylosin	8 (11.3%)	1 (5.9%)

^1^ Number of farms (proportion).

**Table 12:** Multivariable analysis for factors associated with the administration of antibiotics in cases of pneumonia in lambs in sheep flocks in Greece.

Variables	Odds Ratio ^1^ (95% Confidence Intervals)	*p* Value
Family farming tradition		0.043
Yes (59/81 = 72.8% ^2^)	reference	-
No (12/12 = 100.0%)	9.454 (0.537–166.415)	0.12

^1^ Odds ratio calculated against the lowest prevalence associations of the variable. ^2^ Numerator: no. of farms among those included in the denominator, in which the outcome of interest was seen; denominator: no. of farms in which the studied variable prevailed.

**Table 13:** Numbers of small ruminant farms (*n*), on which various antibiotics were administered for the treatment of diarrhoea in lambs or kids in Greece.

Antibiotic	Sheep Flocks (Total *n* = 141)	Goat Herds (Total *n* = 54)
Amoxicillin	35 (24.87%)	13 (24.1%)
Ampicillin	4 (2.8%)	0 (0.0%)
Cephalosporins	3 (2.1%)	0 (0.0%)
Cloxacillin	2 (1.4%)	0 (0.0%)
Colistin	1 (0.7%)	1 (1.9%)
Enrofloxacin	15 (10.6%)	0 (0.0%)
Gentamicin	12 (8.5%)	6 (11.1%)
Lincomycin	4 (2.8%)	2 (3.7%)
Neomycin	2 (1.4%)	0 (0.0%)
Oxytetracycline	46 (32.6%)	19 (35.2%)
Penicillin	25 (17.7%)	11 (20.4%)
Spectinomycin	13 (9.2%)	5 (9.3%)
Streptomycin	17 (12.1%)	5 (9.3%)
Sulfonamides	6 (4.3%)	4 (7.4%)
Tylosin	13 (9.2%)	1 (1.9%)

**Table 14:** Multivariable analysis for factors associated with the administration of antibiotics in cases of diarrhoea in lambs in sheep flocks in Greece.

Variables	Odds Ratio ^1^ (95% Confidence Intervals)	*p* Value
Administration of antibiotics to animals at the dose prescribed		0.017
Yes (136/163 = 83.4% ^2^)	2.470 (1.029–5.930)	0.043
No (22/31 = 71.0%)	reference	-
Routine administration of antibiotics to newborns		0.048
Yes (44/158 = 27.8%)	3.088 (1.032–9.240)	0.044
No (4/36 = 11.1%)	reference	-

^1^ Odds ratios calculated against the lowest prevalence associations of the variable. ^2^ Numerator: no. of farms among those included in the denominator, in which the outcome of interest was seen; denominator: no. of farms in which the studied variable prevailed.

**Table 15:** Summary of multivariable analyses for outcomes about usage of antibiotics on small ruminant farms in Greece.

Outcome	Farm	Variable	*p* Value
Calculation of bodyweight for the administration of antibiotics by weighing	S ^1^	Education of farmers	0.007
		Number of animals on the farms	0.020
	G ^1^	Education of farmers	0.018
Administration of antibiotics to animals at the dose prescribed	S	Number of animals on the farms	0.029
	G	-	>0.37
Observation of the withdrawal period	S	Education of farmer	0.030
	G	-	>0.07
Number of antibiotics used for the treatment of clinical mastitis	S	Professional involvement of farmer	0.011
	G	Professional involvement of farmer	0.015
Administration of antibiotics in cases in abortion	S	Experience of the farmer	0.001
		Season of start of the lambing period	0.007
		Daily period spent by the farmer at the farm	0.010
		Age of farmer	0.012
	G	Age of farmer	0.05
Routine administration of antibiotics to newborns	S	Education of farmer	0.047
	G	-	>0.11
Administration of antibiotics in cases of pneumonia	S	Family farming tradition	0.043
	G	-	>0.15
Administration of antibiotics in cases of diarrhoea	S	Administration of antibiotics at the dose prescribed	0.017
		Routine administration of antibiotics to newborns	0.048
	G	-	>0.10

^1^ S: sheep flocks, G: goat herds.

## Data Availability

Most data presented in this study are in the Supplementary Materials. The remaining data are available on request from the corresponding author. The data are not publicly available as they form part of the PhD thesis of the first author, which has not yet been examined, approved, and uploaded in the official depository of PhD theses from Greek Universities.

## Supplementary Materials

The following supporting information can be downloaded at: https://www.mdpi.com/article/10.3390/antibiotics11060753/s1, Table S1: Details of variables (*n* = 52) collected during interview of farmers by means of a structured questionnaire and used in the evaluations for potential associations with practices of administration of antibiotics in 325 sheep and 119 goat farms during a countrywide investigation in Greece; Table S2: Details of multivariable models (*n* = 14) employed for the evaluation for potential associations with practices of administration of antibiotics on 325 sheep and 119 goat farms during a countrywide investigation in Greece; Table S3: Results of univariable analysis for associations with the method of calculation of bodyweight for the administration of antibiotics on 325 sheep flocks and 119 goat herds in Greece; Table S4: Results of univariable analysis for associations with administration of antibiotics to animals at the dose prescribed on 325 sheep flocks and 119 goat herds in Greece; Table S5: Results of univariable analysis for associations with the observation of the withdrawal period after administration of antibiotics on 325 sheep flocks and 119 goat herds in Greece; Table S6: Results of association of antibiotic used for the treatment of clinical mastitis in accordance with management system applied on the farms and the type of milking, in 325 sheep flocks and 119 goat herds in Greece; Table S7: Association of the pharmaceutical form and number of antibiotic classes used on a farm for the treatment of clinical mastitis with the milk production and quality parameters, as found in a countrywide investigation in Greece; Table S8: Results of univariable analysis for associations of farm–related factors with the number of antibiotics employed for the treatment of clinical mastitis on 325 sheep flocks in Greece; Table S9: Results of univariable analysis for associations of farm–related factors with the number of antibiotics employed for the treatment of clinical mastitis on 119 goat herds in Greece; Table S10: Results of the association of the incidence rate of abortion with the administration of antibiotics in those cases on 325 sheep flocks and 119 goat herds in Greece; Table S11: Results of univariable analysis for associations of farm-related factors with the use of antibiotics in cases of abortion on 325 sheep flocks in Greece; Table S12: Results of univariable analysis for associations of farm-related factors with the use of antibiotics in cases of abortion on 119 goat herds in Greece; Table S13: Results of univariable analysis for associations with the routine administration of antibiotics to newborn lambs/kids on 325 sheep flocks and 119 goat herds in Greece; Table S14: Incidence rate of lamb/kid pneumonia or diarrhoea on 325 sheep flocks and 119 goat herds in Greece, in accordance with the routine administration of antibiotics to newborns; Table S15: Results of the association of the incidence rate of lamb/kid pneumonia with the administration of antibiotics in those cases on 325 sheep flocks and 119 goat herds in Greece; Table S16: Results of univariable analysis for associations of farm-related factors with the use of antibiotics in cases of lamb pneumonia on 325 sheep flocks in Greece; Table S17: Results of univariable analysis for associations of farm-related factors with the use of antibiotics in cases of kid pneumonia on 119 goat herds in Greece; Table S18: Results of the association of the incidence rate of lamb/kid diarrhoea with the administration of antibiotics in those cases on 325 sheep flocks and 119 goat herds in Greece; Table S19: Results of univariable analysis for associations of farm-related factors with the use of antibiotics in cases of lamb diarrhoea on 325 sheep flocks in Greece; Table S20: Results of univariable analysis for associations of farm-related factors with the use of antibiotics on cases of kid diarrhoea in 119 goat herds in Greece.
