# Supplementary material for: Use of Antibiotics against Bacterial Infections on Dairy Sheep and Goat Farms: Patterns of Usage and Associations with Health Management and Human Resources
Source: Antibiotics (Basel). 2022 May 31;11(6):753. doi: 10.3390/antibiotics11060753 (PMC9219691; doi:10.3390/antibiotics11060753)
Supplement: Supplementary file 1 [file antibiotics-11-00753-s001.zip › antibiotics-1755049-supplementary.pdf]

Use of Antibiotics Against Bacterial Infections on Dairy Sheep and Goat Farms: Patterns of Usage and Associations with Health Management and Human Resources

Daphne T. Lianou and George C. Fthenakis

Table S1. Details of variables (n = 52) collected during interview of farmers by means of a structured questionnaire and used in the evaluations for potential associations with patterns of usage of antibiotics in 444 small ruminant farms during a countrywide investigation in Greece.

Management system applied in the farm (description according to EFSA classification [1])
Type of milking (hand-milking / machine-milking)
No. of female animals in the farm (no.)
Breed of animals in the farm (description)
Average age of culling female animals (years)
Month of the start of the lambing / kidding season (description)
Total milk quantity per ewe / doe obtained during the preceding milking period (litres)
Total number of lambs / kids born during the preceding lambing season (no.)
Collaboration with a veterinarian (yes / no)
Means of calculating live bodyweight for the administration of antibiotics (weighing / estimation)
Routine administration of antibiotics at the dose prescribed (yes / no)
Use of laboratory diagnostic examinations in samples of milk (yes / no)
Observation of the prescribed withdrawal periods after administration of antibiotics (yes / no)
Total cases of clinical mastitis during the preceding season (no.)
Treatment performed (yes / no)
Pharmaceuticals used for treatment (description)
Route for administration of antimicrobials (systemically / intramammary)
Total cases of abortion during the preceding season (no.)
Treatment performed (yes / no)
Pharmaceuticals used for treatment (description)
Total cases of lamb / kid pneumonia during the preceding season (no.)
Treatment performed (yes / no)
Pharmaceuticals used for treatment (description)
Total cases of lamb / kid diarrhoea during the preceding season (no.)
Treatment performed (yes / no)
Pharmaceuticals used for treatment (description)
Induction of lambing (yes / no)
Newborn care and specific monitoring (yes / no)
Maintenance of a colostrum bank (yes / no)

Newborn fostering to female animals other than their dams (yes / no)

Administration of ‘dry-ewe’ treatment at the end of the lactation period (yes / no)

Daily number of milking sessions (no.)

Vaccination against *Chlamydia* infection (yes / no)

Vaccination against *Toxoplasma* infection (yes / no)

Vaccination against *Brucella* infection (yes / no)

Vaccination against clostridial infection (yes / no)

Vaccination against bacterial mastitis (yes / no)

Vaccination against contagious agalactia (yes / no)

Vaccination against bacterial respiratory infections (yes / no)

Administration of selenium to pregnant animals (yes / no)

Administration of selenium to newborn animals (yes / no)

Disinfection of the navel stump in newborns (yes / no)

Tail docking in newborns (yes / no)

Routine administration of antibiotics to newborns (yes / no)

Antimicrobials administered (description)

Age of farmer (years)

Length of previous animal farming experience (years)

Farmer’s general education (description: primary = European Qualifications Framework Levels 1 or 2, secondary = or post-secondary = European Qualifications Framework Levels 3, 4 or 5, tertiary = European Qualifications Framework Level 6, 7 or 8)

Farmer’s professional involvement in farming (full-time / part-time)

Daily period spent by farmer at the farm (hours)

Family tradition in farming (yes / no)

Presence of working staff in the farm (yes/no)

---

[1] management system classified as intensive, semi-intensive, semi-extensive, extensive (European Food Safety Authority. Scientific opinion on the welfare risks related to the farming of sheep for wool, meat and milk production. *EFSA J.* **2014**, *12*, 3933–4060.).

Table S2. Details of multivariable models (n = 16) employed for the evaluation for potential associations with practices of administration of antibiotics on 325 sheep and 119 goat farms during a countrywide investigation in Greece.

Outcome	Variables		
	**assessed in univariable analyses (*n*)**	**offered to the multivariable models (*n*)**	**required in the final models**
Calculation of bodyweight for the administration of antibiotics by weighing – sheep	10	6	(a) Management system applied in the farm, (b) No. of female animals in the farm, (c) Collaboration with a veterinarian, (d) Farmer's general education
Calculation of bodyweight for the administration of antibiotics by weighing – goats	10	2	(a) Management system applied in the farm, (b) Farmer's general education
Administration of antibiotics to animals at the dose prescribed – sheep	10	2	(a) No. of female animals in the farm, (b) Collaboration with a veterinarian
Administration of antibiotics to animals at the dose prescribed – goats	10	2	(a) Management system applied in the farm, (b) Farmer's professional involvement in farming
Observation of the withdrawal period after administration of antibiotics – sheep	10	2	(a) No. of female animals in the farm, (b) Farmer's general education
Observation of the withdrawal period after administration of antibiotics – goats	10	1	(a) Farmer's professional involvement in farming
Number of antibiotics used for the treatment of clinical mastitis – sheep	21	4	(a) No. of female animals in the farm, (b) Farmer's age, (c) Farmer's professional involvement in farming
Number of antibiotics used for the treatment of clinical mastitis – goats	21	2	(a) Farmer's professional involvement in farming
Use of antibiotics in cases of abortion – sheep	18	10	(a) Month of the start of the lambing / kidding season, (b) Means of calculating live bodyweight for the administration of antibiotics, (c) Farmer's age, (d) Length of previous animal farming experience, (e) Daily period spent by farmer at the farm
Use of antibiotics in cases of abortion – goats	18	5	(a) Collaboration with a veterinarian, (b) Administration of antibiotics to animals at the dose prescribed, (c) Farmer's age, (d) Presence of working staff in the farm

Routine administration of antibiotics to newborns - sheep	10	3	(a) Collaboration with a veterinarian, (b) Farmer’s age, (c) Farmer’s general education
Routine administration of antibiotics to newborns - goats	10	3	(a) Management system applied in the farm, (b) Collaboration with a veteri-narian
Use of antibiotics for the treatment of pneumonia– sheep	25	7	(a) Start of lambing period, (b) Means of calculating live bodyweight for the administration of antibiotics, (c) Routine administration of antibiotics to newborns, (d) Daily peri-od spent by farmer at the farm, (e) Family tradition in farming
Use of antibiotics for the treatment of pneumonia– goats	25	4	(a) Means of calculating live body-weight for the administration of antibiotics, (b) Farmer’s age
Use of antibiotics for the treatment of diarrhoea– sheep	25	5	(a) Collaboration with a veterinarian, (b) Administration of antibiotics to animals at the dose prescribed (c) Routine administration of antibiotics to newborns, (d) Presence of working staff in the farm
Use of antibiotics for the treatment of diarrhoea– goats	25	5	(a) Breed of animals in the farm, (b) Newborn fostering to female animals other than their dam, (c) Routine administration of antibiotics to newborns

Table S3. Results of univariable analysis for associations with the method of calculation of bodyweight for the administration of antibiotics on 325 sheep flocks and 119 goat herds in Greece.

(a) Sheep flocks

Estimation (n = 252)				Weighing (n = 73)				p
**Management system applied in the farm**								
Intensive	Semi-intensive	Semi-extensive	Extensive	Intensive	Semi-intensive	Semi-extensive	Extensive	
33	109	102	8	11	31	14	17	< 0.0001
**No. of ewes in the flock**								
≤ 165 ewes	166-330 ewes	331-500 ewes	> 500 ewes	≤ 165 ewes	166-330 ewes	331-500 ewes	> 500 ewes	
75	94	46	37	13	26	20	14	0.11
**Collaboration with a veterinarian**								
Yes	No			Yes	No			
225	27			58	15			0.027
**Age of the farmer**								
Up to 50 years	Over 50 years			Up to 50 years	Over 50 years			
158	94			39	34			0.15
**Length of previous animal farming experience of the farmer**								
≤ 5 years	> 5 years			≤ 5 years	> 5 years			
62	190			12	61			0.15
**Education of the farmer**								
Primary education	Secondary or post-secondary education	Tertiary education		Primary education	Secondary education	Tertiary education		
54	167	31		3	58	12		0.003
**Professional involvement in farming**								
Full-time	Part-time			Full-time	Part-time			
227	25			65	8			0.80
**Daily period at the farm**								
≤ 8 hours	> 8 hours			≤ 8 hours	> 8 hours			
79	173			20	53			0.52
**Family tradition in farming**								
Yes	No			Yes	No			
218	34			65	8			0.57

**Presence of working staff in the flock**				
Yes	No	Yes	No	
100	152	23	50	0.21

(b) Goat herds

**Estimation (*n* = 93)**				**Weighing (*n* = 26)**				***p***
**Management system applied in the farm**								
Intensive	Semi-intensive	Semi-extensive	Extensive	Intensive	Semi-intensive	Semi-extensive	Extensive	
9	20	52	12	0	9	9	8	0.022
**No. of does in the herd**								
≤ 165 ewes	166-330 ewes	331-500 ewes	> 500 ewes	≤ 165 ewes	166-330 ewes	331-500 ewes	> 500 ewes	
42	32	9	10	14	5	4	3	0.48
**Collaboration with a veterinarian**								
Yes		No		Yes		No		
80		13		21		5		0.51
**Age of the farmer**								
Up to 50 years		Over 50 years		Up to 50 years		Over 50 years		
58		35		15		11		0.66
**Length of previous animal farming experience of the farmer**								
≤ 5 years		> 5 years		≤ 5 years		> 5 years		
19		74		5		21		0.89
**Education of the farmer**								
Primary education	Secondary or post-secondary education	Tertiary education		Primary education	Secondary education	Tertiary education		
20	66	7		0	23	3		0.033
**Professional involvement in farming**								
Full-time		Part-time		Full-time		Part-time		
81		12		24		2		0.47
**Daily period at the farm**								
≤ 8 hours		> 8 hours		≤ 8 hours		> 8 hours		
22		71		6		20		0.95
**Family tradition in farming**								
Yes		No		Yes		No		
81		12		22		4		0.74

**Presence of working staff in the herd**				
Yes	No	Yes	No	
28	65	6	20	0.48

Table S4. Results of univariable analysis for associations with administration of antibiotics to animals at the dose prescribed on 325 sheep flocks and 119 goat herds in Greece.

(a) Sheep flocks

**Administration at a higher dose than that prescribed (*n* = 61)**				**Administration at the dose prescribed (*n* = 264)**				***p***
**Management system applied in the farm**								
Intensive	Semi-intensive	Semi-extensive	Extensive	Intensive	Semi-intensive	Semi-extensive	Extensive	
7	32	16	6	37	108	100	19	0.25
**No. of ewes in the flock**								
≤ 165 ewes	166-330 ewes	331-500 ewes	> 500 ewes	≤ 165 ewes	166-330 ewes	331-500 ewes	> 500 ewes	
9	24	16	12	79	96	50	39	0.09
**Collaboration with a veterinarian**								
Yes		No		Yes		No		
48		13		234		30		0.039
**Age of the farmer**								
Up to 50 years		Over 50 years		Up to 50 years		Over 50 years		
40		21		157		107		0.38
**Length of previous animal farming experience of the farmer**								
≤ 5 years		> 5 years		≤ 5 years		> 5 years		
16		45		58		206		0.47
**Education of the farmer**								
Primary education	Secondary or post-secondary education	Tertiary education		Primary education	Secondary education	Tertiary education		
11	38	12		46	187	31		0.24
**Professional involvement in farming**								
Full-time		Part-time		Full-time		Part-time		
54		7		238		26		0.70
**Daily period at the farm**								
≤ 8 hours		> 8 hours		≤ 8 hours		> 8 hours		
16		45		83		181		0.43
**Family tradition in farming**								
Yes		No		Yes		No		
54		7		229		35		0.71
**Presence of working staff in the flock**								
Yes		No		Yes		No		
22		39		101		163		0.75

(b) Goat herds

Administration at a higher dose than that prescribed (*n* = 26)				Administration at the dose prescribed (*n* = 93)				*p*
**Management system applied in the farm**								
Intensive	Semi-intensive	Semi-extensive	Extensive	Intensive	Semi-intensive	Semi-extensive	Extensive	
0	9	11	6	9	20	50	14	0.15
**No. of does in the herd**								
≤ 165 ewes	166-330 ewes	331-500 ewes	> 500 ewes	≤ 165 ewes	166-330 ewes	331-500 ewes	> 500 ewes	
12	7	3	4	44	30	10	9	0.85
**Collaboration with a veterinarian**								
Yes	No			Yes	No			
22	4			79	14			0.97
**Age of the farmer**								
Up to 50 years	Over 50 years			Up to 50 years	Over 50 years			
16	10			57	36			0.98
**Length of previous animal farming experience of the farmer**								
≤ 5 years	> 5 years			≤ 5 years	> 5 years			
4	22			20	73			0.49
**Education of the farmer**								
Primary education	Secondary or post-secondary education	Tertiary education		Primary education	Secondary education	Tertiary education		
7	17	2		13	72	8		0.30
**Professional involvement in farming**								
Full-time	Part-time			Full-time	Part-time			
21	5			84	9			0.18
**Daily period at the farm**								
≤ 8 hours	> 8 hours			≤ 8 hours	> 8 hours			
6	20			22	71			0.95
**Family tradition in farming**								
Yes	No			Yes	No			
22	4			81	12			0.74
**Presence of working staff in the herd**								
Yes	No			Yes	No			
6	20			28	65			0.48

Table S5. Results of univariable analysis for associations with the observation of the withdrawal period after administration of antibiotics on 325 sheep flocks and 119 goat herds in Greece.

(a) Sheep flocks

**No observation (*n* = 4)**				**Observation (*n* = 321)**				***p***
**Management system applied in the farm**								
Intensive	Semi-intensive	Semi-extensive	Extensive	Intensive	Semi-intensive	Semi-extensive	Extensive	
0	1	3	0	44	139	113	25	0.40
**No. of ewes in the flock**								
≤ 165 ewes	166-330 ewes	331-500 ewes	> 500 ewes	≤ 165 ewes	166-330 ewes	331-500 ewes	> 500 ewes	
0	1	3	0	87	117	66	51	0.06
**Collaboration with a veterinarian**								
Yes	No			Yes	No			
4	0			279	42			0.44
**Age of the farmer**								
Up to 50 years	Over 50 years			Up to 50 years	Over 50 years			
3	1			194	127			0.55
**Length of previous animal farming experience of the farmer**								
≤ 5 years	> 5 years			≤ 5 years	> 5 years			
0	4			74	247			0.27
**Education of the farmer**								
Primary education	Secondary or post-secondary education	Tertiary education		Primary education	Secondary education	Tertiary education		
1	1	2		56	224	41		0.07
**Professional involvement in farming**								
Full-time	Part-time			Full-time	Part-time			
4	0			288	33			0.50
**Daily period at the farm**								
≤ 8 hours	> 8 hours			≤ 8 hours	> 8 hours			
2	2			97	224			0.39
**Family tradition in farming**								
Yes	No			Yes	No			
4	0			279	42			0.44

**Presence of working staff in the flock**				
Yes	No	Yes	No	
1	3	122	199	0.59

(b) Goat herds

**No observation (*n* = 2)**				**Observation (*n* = 117)**				***p***
**Management system applied in the farm**								
Intensive	Semi-intensive	Semi-extensive	Extensive	Intensive	Semi-intensive	Semi-extensive	Extensive	
0	0	1	1	9	29	60	19	0.58
**No. of does in the herd**								
≤ 165 ewes	166-330 ewes	331-500 ewes	> 500 ewes	≤ 165 ewes	166-330 ewes	331-500 ewes	> 500 ewes	
1	0	0	1	55	37	13	12	0.30
**Collaboration with a veterinarian**								
Yes		No		Yes		No		
2		0		99		18		0.55
**Age of the farmer**								
Up to 50 years		Over 50 years		Up to 50 years		Over 50 years		
2		0		71		46		0.26
**Length of previous animal farming experience of the farmer**								
≤ 5 years		> 5 years		≤ 5 years		> 5 years		
1		1		23		94		0.29
**Education of the farmer**								
Primary education	Secondary or post-secondary education	Tertiary education		Primary education	Secondary education	Tertiary education		
0	2	0		20	87	10		0.71
**Professional involvement in farming**								
Full-time		Part-time		Full-time		Part-time		
1		1		104		13		0.09
**Daily period at the farm**								
≤ 8 hours		> 8 hours		≤ 8 hours		> 8 hours		
0		2		28		89		0.43
**Family tradition in farming**								
Yes		No		Yes		No		
2		0		101		16		0.57

**Presence of working staff in the herd**				
Yes	No	Yes	No	
0	2	34	83	0.37

Table S6. Results of association of antibiotics used for the treatment of clinical mastitis in accordance with management system applied on the farms and the type of milking, on 325 sheep flocks and 119 goat herds in Greece.

(a) Sheep flocks

	Amoxi-cillin	Ampi-cillin	Cephalo-sporins	Cloxa-cillin	Enroflo-xacin	Flor-fenicol	Genta-micin	Linco-mycin	Marbo-floxacin	Oxytetra-cycline	Peni-cillin	Spectin-omycin	Strepto-mycin	Tylosin
**Number of flocks in which respective antibiotics were used**														
Antibiotics														
Management system applied in the farms ($p = 0.42$)														
Intensive	3	0	3	1	1	0	0	0	2	7	27	0	24	4
Semi-intensive	6	1	2	3	5	1	3	4	3	27	90	2	83	1
Semi-extensive	2	0	3	0	1	1	1	3	1	17	82	2	78	6
Extensive	0	0	2	0	0	0	0	0	0	9	19	0	15	0
Type of milking ($p = 0.81$)														
Machine-milking	9	1	8	3	6	2	3	6	6	45	165	4	152	11
Hand-milking	2	0	2	1	1	0	1	1	0	15	53	0	48	0

(b) Goat herds

	Amoxi-cillin	Ampi-cillin	Cephalo-sporins	Cloxa-cillin	Enroflo-xacin	Flor-fenicol	Genta-micin	Linco-mycin	Marbo-floxacin	Oxytetra-cycline	Peni-cillin	Spectin-omycin	Strepto-mycin	Tylosin
**Number of herds in which respective antibiotics were used**														
Antibiotics														
Management system applied in the farms ($p = 0.92$)														
Intensive	0	0	0	0	1	0	0	0	0	3	5	0	5	1
Semi-intensive	1	1	0	1	1	0	0	1	0	7	11	0	10	0
Semi-extensive	2	0	1	1	1	0	0	2	0	10	29	2	27	3
Extensive	0	0	0	1	0	0	0	0	0	0	8	0	8	0
Type of milking ($p = 0.18$)														
Machine-milking	2	1	1	3	3	0	0	3	0	12	28	2	26	4
Hand-milking	1	0	0	0	0	0	0	0	0	8	25	0	24	0

Table S7. Association of the pharmaceutical form and number of antibiotic classes used in a farm for the treatment of clinical mastitis with the milk production and quality parameters, as found in a countrywide investigation in Greece.

(a) Pharmaceutical form used

**Pharmaceutical form used**	**Bulk-tank milk somatic cell counts (× 10^6 cells mL^{-1})**		**Total bacterial counts (× 10^3 cfu mL^{-1})**		**Isolation of staphylococci from bulk-tank milk (% of farms)**		**Fat content (%)**		**Protein content (%)**		**Yearly milk production (L per animal)**	
	Sheep flocks	Goat herds	Sheep flocks	Goat herds	Sheep flocks	Goat herds	Sheep flocks	Goat herds	Sheep flocks	Goat herds	Sheep flocks	Goat herds
Injectable (*n* = 240 / 63 [1])	0.501 (0.455 – 0.552) [3]	0.922 (0.814 – 1.044)	407 (339 – 490)	646 (427 – 977)	62.9%	65.1%	6.18 ± 0.06 [4]	4.79 ± 0.14	4.43 ± 0.02	3.23 ± 0.04	203 ± 5	218 ± 17
For i/m [2] administration (*n* = 19 / 8)	0.393 (0.296 – 0.522)	0.764 (0.541 – 1.081)	309 (117 – 813)	251 (78 – 813)	63.2%	75.0%	5.96 ± 0.16	4.95 ± 0.56	4.44 ± 0.07	3.23 ± 0.09	234 ± 21	209 ± 36
Injectable & for i/m administration (n = 11 / 0)	0.743 (0.487 – 1.135)	-	1,413 (309 – 6,457)	-	81.8%	-	6.05 ± 0.10	-	4.45 ± 0.06	-	216 ± 30	-
p	0.07	0.33	0.035	0.13	0.44	0.58	0.52	0.71	0.95	0.93	0.27	0.86

(b) Number of antibiotics used

**Number of antibiotics used**	**Bulk-tank milk somatic cell counts (× 10^6 cells mL^{-1})**		**Total bacterial counts (× 10^3 cfu mL^{-1})**		**Isolation of staphylococci from bulk-tank milk (% of farms)**		**Fat content (%)**		**Protein content (%)**		**Yearly milk production (L per animal)**	
	Sheep flocks	Goat herds	Sheep flocks	Goat herds	Sheep flocks	Goat herds	Sheep flocks	Goat herds	Sheep flocks	Goat herds	Sheep flocks	Goat herds
1 (*n* = 52 / 15 [1])	0.439 (0.367 – 0.526)	0.823 (0.621 – 1.096)	324 (204 – 513)	490 (224 – 1,072)	63.5%	53.3%	6.13 ± 0.14	4.41 ± 0.30	4.39 ± 0.05	3.17 ± 0.07	207 ± 11	228 ± 32
2 (*n* = 169 / 40)	0.537 (0.480 – 0.600)	0.974 (0.836 – 1.135	427 (339 – 537)	724 (479 – 1,096)	62.1%	70.0%	6.15 ± 0.06	5.05 ± 0.17	4.44 ± 0.02	3.23 ± 0.04	201 ± 7	211 ± 17
≥ 3 (*n* = 49 / 16)	0.458 (0.364 – 0.575)	0.825 (0.652 – 1.044)	537 (309 – 933)	407 (123 – 1,349)	69.4%	68.8%	6.21 ± 0.13	4.55 ± 0.32	4.45 ± 0.03	3.33 ± 0.10	218 ± 12	223 ± 48
p	0.16	0.41	0.30	0.47	0.65	0.49	0.88	0.11	0.45	0.31	0.45	0.90

[1] number of sheep / goat farms.

[2] intramammary.

[3] mean (95% confidence intervals).

[4] mean ± standard error of the mean.

Table S8. Results of univariable analysis for associations of farm-related factors with the number of antibiotics used for the treatment of clinical mastitis on 325 sheep flocks in Greece.

**Management system applied in the farm**				
Intensive	Semi-intensive	Semi-extensive	Extensive	
2.24 ± 0.15	1.96 ± 0.06	2.01 ± 0.07	2.25 ± 0.12	0.11
**Type of milking**				
Machine-milking	Hand-milking			
2.00 ± 0.75	2.14 ± 0.07			0.21
**No. of ewes in the flock**				
≤ 165 ewes	166-330 ewes	331-500 ewes	> 500 ewes	
2.15 ± 0.09	1.97 ± 0.07	2.17 ± 0.10	1.81 ± 0.10	0.030
**Breed of animals**				
Crossbreeds	Imported breeds	Local breeds		
1.97 ± 0.07	1.99 ± 0.07	2.09 ± 0.07		0.48
**Average age of culling ewes**				
≤ 6 years	> 6 years			
2.01 ± 0.05	2.10 ± 0.07			0.31
**Start of lambing period**				
All year	Autumn	Winter	Spring - Summer	
2.00 ± 0.10	1.96 ± 0.06	2.10 ± 0.06	2.33 ± 0.42	0.32
**Collaboration with a veterinarian**				
Yes	No			
2.02 ± 0.05	2.13 ± 0.12			0.47
**Method of calculation of bodyweight for the administration of antibiotics**				
Estimation	Weighing			
2.02 ± 0.05	1.98 ± 0.10			0.54
**Administration of antibiotics to animals at the dose prescribed**				
Administration at a higher dose than that prescribed	Administration at the dose prescribed			
2.07 ± 0.10	2.02 ± 0.05			0.64
**Use of preventive diagnostic examinations in milk**				
Yes	No			
1.94 ± 0.06	2.06 ± 0.05			0.22
**Use of 'dry-period' treatment**				
Yes	No			
2.14 ± 0.12	2.01 ± 0.05			0.24

**Daily number of milking sessions**			
One or two	Three		
2.01 ± 0.05	2.12 ± 0.09		0.34
**Vaccination against bacterial mastitis**			
Yes	No		
2.03 ± 0.07	2.04 ± 0.06		0.90
**Vaccination against contagious agalactia**			
Yes	No		
2.04 ± 0.06	2.02 ± 0.06		0.76
**Age of the farmer**			
≤ 50 years	> 50 years		
1.98 ± 0.05	2.13 ± 0.08		0.09
**Length of previous animal farming experience of the farmer**			
≤ 5 years	> 5 years		
2.07 ± 0.09	2.02 ± 0.05		0.69
**Education of the farmer**			
Primary education	Secondary or post-secondary education	Tertiary education	
1.94 ± 0.09	2.03 ± 0.05	2.20 ± 0.14	0.25
**Professional involvement in farming**			
Full-time	Part-time		
2.07 ± 0.05	1.73 ± 0.12		0.023
**Daily period at the farm**			
≤ 8 hours	> 8 hours		
2.08 ± 0.08	2.01 ± 0.05		0.43
**Family tradition in farming**			
Yes	No		
2.03 ± 0.05	2.09 ± 0.14		0.85
**Presence of working staff in the flock**			
Yes	No		
2.04 ± 0.07	2.03 ± 0.05		0.92

Table S9. Results of univariable analysis for associations of farm-related factors with the number of antibiotics used for the treatment of clinical mastitis on 119 goat herds in Greece.

**Management system applied in the farm**				
Intensive	Semi-intensive	Semi-extensive	Extensive	
2.50 ± 0.34	1.65 ± 0.13	2.08 ± 0.12	2.33 ± 0.24	0.020
**Type of milking**				
Machine-milking	Hand-milking			
2.07 ± 0.12	2.03 ± 0.12			0.83
**No. of does in the herd**				
≤ 165 ewes	166-330 ewes	331-500 ewes	> 500 ewes	
2.03 ± 0.13	2.05 ± 0.16	2.43 ± 0.37	1.89 ±0.20	0.54
**Breed of animals**				
Crossbreeds	Imported breeds	Local breeds		
2.11 ± 0.31	2.03 ± 0.17	2.06 ± 0.10		0.97
**Average age of culling does**				
≤ 6 years	> 6 years			
2.00 ± 0.14	2.09 ± 0.12			0.64
**Start of kidding period**				
All year	Autumn	Winter	Spring - Summer	
2.40 ± 0.40	2.13 ± 0.16	1.98 ± 0.12	2.00	0.44
**Collaboration with a veterinarian**				
Yes	No			
2.10 ± 0.10	1.82 ± 0.18			0.26
**Method of calculation of bodyweight for the administration of antibiotics**				
Estimation	Weighing			
2.09 ± 0.10	1.93 ± 0.21			0.48
**Administration of antibiotics to animals at the dose prescribed**				
Administration at a higher dose than that prescribed	Administration at the dose prescribed			
2.00 ± 0.22	2.07 ± 0.10			0.74
**Use of preventive diagnostic examinations in milk**				
Yes	No			
2.06 ± 0.06	2.05 ± 0.11			0.97
**Use of 'dry-period' treatment**				
Yes	No			
2.13 ± 0.30	2.05 ± 0.09			0.79

**Daily number of milking sessions**			
One or two	Three		
2.07 ± 0.09	1.75 ± 0.25		0.41
**Vaccination against bacterial mastitis**			
Yes	No		
2.00± 0.15	2.09 ± 0.11		0.66
**Vaccination against contagious agalactia**			
Yes	No		
2.12 ± 0.12	1.97 ± 0.13		0.40
**Age of the farmer**			
≤ 50 years	> 50 years		
2.03 ± 0.12	2.10 ± 0.13		0.69
**Length of previous animal farming experience of the farmer**			
≤ 5 years	> 5 years		
1.93 ± 0.21	2.09 ± 0.10		0.48
**Education of the farmer**			
Primary education	Secondary or post-secondary education	Tertiary education	
2.08 ± 0.23	2.12 ± 0.10	1.57 ± 0.30	0.21
**Professional involvement in farming**			
Full-time	Part-time		
2.14 ± 0.09	1.38 ± 0.26		0.006
**Daily period at the farm**			
≤ 8 hours	> 8 hours		
2.07 ± 0.21	2.05 ± 0.10		0.95
**Family tradition in farming**			
Yes	No		
2.05 ± 0.09	2.11 ± 0.30		0.82
**Presence of working staff in the flock**			
Yes	No		
2.07 ± 0.17	2.04 ± 0.10		0.81

Table S10. Results of the association of the incidence rate of abortion with the administration of antibiotics in those cases on 325 sheep flocks and 119 goat herds in Greece.

Incidence rate	Sheep flocks		Goat herds	
	Administration of antibiotics in cases of abortion	No administration of antibiotics in cases of abortion	Administration of antibiotics in cases of abortion	No administration of antibiotics in cases of abortion
≤ 2%	22 [1]	33	10	2
> 2%	46	53	29	16
p	0.44		0.21	

[1] number of sheep / goat farms.

Table S11. Results of univariable analysis for associations of farm-related factors with the use of antibiotics in cases of abortion on 325 sheep flocks in Greece.

Use of antibiotics (*n* = 68)				No use of antibiotics (*n* = 86)				*p*
**Management system applied in the farm**								
Intensive	Semi-intensive	Semi-extensive	Extensive	Intensive	Semi-intensive	Semi-extensive	Extensive	
11	30	24	3	12	34	31	9	0.55
**No. of ewes in the flock**								
≤ 165 ewes	166-330 ewes	331-500 ewes	> 500 ewes	≤ 165 ewes	166-330 ewes	331-500 ewes	> 500 ewes	
15	25	17	11	23	32	16	15	0.78
**Breed of animals**								
Crossbreeds	Imported breeds	Local breeds		Crossbreeds	Imported breeds	Local breeds		
11	34	23		6	33	47		0.021
**Average age of culling ewes**								
≤ 6 years	> 6 years			≤ 6 years	> 6 years			
49	19			52	34			0.13
**Start of lambing period**								
All year	Autumn	Winter	Spring - Summer	All year	Autumn	Winter	Spring - Summer	
3	44	20	1	6	31	46	3	0.007
**Collaboration with a veterinarian**								
Yes	No			Yes	No			
60	8			73	13			0.55
**Method of calculation of bodyweight for the administration of antibiotics**								
Estimation	Weighing			Estimation	Weighing			
62	6			65	21			0.011
**Administration of antibiotics to animals at the dose prescribed**								
Administration at a higher dose than that prescribed	Administration at the dose prescribed			Administration at a higher dose than that prescribed	Administration at the dose prescribed			
16	52			18	68			0.70
**Vaccination against *Chlamydia* infection**								
Yes	No			Yes	No			
32	36			29	57			0.09
**Vaccination against *Toxoplasma* infection**								
Yes	No			Yes	No			
0	68			0	86			n/a

**Vaccination against *Brucella* infection**						
Yes	No		Yes	No		
68	0		79	7		0.016
**Age of the farmer**						
Up to 50 years	Over 50 years		Up to 50 years	Over 50 years		
51	17		47	39		0.009
**Length of previous animal farming experience of the farmer**						
≤ 5 years	> 5 years		≤ 5 years	> 5 years		
12	56		29	57		0.025
**Education of the farmer**						
Primary education	Secondary or post-secondary education	Tertiary education	Primary education	Secondary education	Tertiary education	
17	42	8	12	65	9	0.17
**Professional involvement in farming**						
Full-time	Part-time		Full-time	Part-time		
65	3		78	8		0.24
**Daily period at the farm**						
≤ 8 hours	> 8 hours		≤ 8 hours	> 8 hours		
13	55		33	53		0.01
**Family tradition in farming**						
Yes	No		Yes	No		
60	8		70	16		0.25
**Presence of working staff in the flock**						
Yes	No		Yes	No		
25	43		34	52		0.73

Table S12. Results of univariable analysis for associations of farm-related factors with the use of antibiotics in cases of abortion on 119 goat herds in Greece.

**Use of antibiotics (*n* = 39)**				**No use of antibiotics (*n* = 18)**				***p***
**Management system applied in the farm**								
Intensive	Semi-intensive	Semi-extensive	Extensive	Intensive	Semi-intensive	Semi-extensive	Extensive	
4	9	19	7	2	3	12	1	0.51
**No. of does in the herd**								
≤ 165 ewes	166-330 ewes	331-500 ewes	> 500 ewes	≤ 165 ewes	166-330 ewes	331-500 ewes	> 500 ewes	
15	15	2	7	11	4	1	2	0.44
**Breed of animals**								
Crossbreeds	Imported breeds	Local breeds		Crossbreeds	Imported breeds	Local breeds		
4	18	17		5	3	10		0.06
**Average age of culling does**								
≤ 6 years	> 6 years			≤ 6 years	> 6 years			
15	24			4	14			0.23
**Start of kidding period**								
All year	Autumn	Winter	Spring - Summer	All year	Autumn	Winter	Spring - Summer	
2	17	19	1	2	5	11	0	0.52
**Collaboration with a veterinarian**								
Yes	No			Yes	No			
31	8			17	1			0.15
**Method of calculation of bodyweight for the administration of antibiotics**								
Estimation	Weighing			Estimation	Weighing			
34	5			15	3			0.70
**Administration of antibiotics to animals at the dose prescribed**								
Administration at a higher dose than that prescribed	Administration at the dose prescribed			Administration at a higher dose than that prescribed	Administration at the dose prescribed			
10	29			1	17			0.07
**Vaccination against *Chlamydia* infection**								
Yes	No			Yes	No			
13	26			5	13			0.67
**Vaccination against *Toxoplasma* infection**								
Yes	No			Yes	No			
0	39			0	18			n/a

**Vaccination against *Brucella* infection**				
Yes	No	Yes	No	
37	2	17	1	0.95
**Age of the farmer**				
Up to 50 years	Over 50 years	Up to 50 years	Over 50 years	
28	11	8	10	0.047
**Length of previous animal farming experience of the farmer**				
≤ 5 years	> 5 years	≤ 5 years	> 5 years	
7	32	5	13	0.40

**Education of the farmer**						
Primary education	Secondary or post-secondary education	Tertiary education	Primary education	Secondary education	Tertiary education	
10	26	3	3	13	2	0.72

**Professional involvement in farming**				
Full-time	Part-time	Full-time	Part-time	
35	4	17	1	0.56
**Daily period at the farm**				
≤ 8 hours	> 8 hours	≤ 8 hours	> 8 hours	
10	29	6	12	0.55
**Family tradition in farming**				
Yes	No	Yes	No	
34	5	14	4	0.37
**Presence of working staff in the flock**				
Yes	No	Yes	No	
13	26	3	15	0.19

Table S13. Results of univariable analysis for associations with the routine administration of antibiotics to newborn lambs / kids on 325 sheep flocks and 119 goat herds in Greece.

(a) Sheep flocks

**No routine administration (*n* = 260)**				**Routine administration (*n* = 65)**				***p***
**Management system applied in the farm**								
Intensive	Semi-intensive	Semi-extensive	Extensive	Intensive	Semi-intensive	Semi-extensive	Extensive	
32	112	94	22	12	28	22	3	0.47
**No. of ewes in the flock**								
≤ 165 ewes	166-330 ewes	331-500 ewes	> 500 ewes	≤ 165 ewes	166-330 ewes	331-500 ewes	> 500 ewes	
66	97	57	40	22	23	9	11	0.37
**Collaboration with a veterinarian**								
Yes	No			Yes	No			
222	38			61	4			0.07
**Age of the farmer**								
Up to 50 years	Over 50 years			Up to 50 years	Over 50 years			
152	108			45	20			0.11
**Length of previous animal farming experience of the farmer**								
≤ 5 years	> 5 years			≤ 5 years	> 5 years			
59	201			15	50			0.95
**Education of the farmer**								
Primary education	Secondary or post-secondary education	Tertiary education		Primary education	Secondary education	Tertiary education		
36	191	33		21	34	10		0.001
**Professional involvement in farming**								
Full-time	Part-time			Full-time	Part-time			
233	27			59	6			0.78
**Daily period at the farm**								
≤ 8 hours	> 8 hours			≤ 8 hours	> 8 hours			
78	182			21	44			0.72
**Family tradition in farming**								
Yes	No			Yes	No			
225	235			58	7			0.56
**Presence of working staff in the flock**								
Yes	No			Yes	No			
98	162			25	40			0.91

(b) Goat herds

**No routine administration (n = 90)**				**Routine administration (n = 29)**				***p***
**Management system applied in the farm**								
Intensive	Semi-intensive	Semi-extensive	Extensive	Intensive	Semi-intensive	Semi-extensive	Extensive	
6	20	45	19	3	9	16	1	0.15
**No. of does in the herd**								
≤ 165 ewes	166-330 ewes	331-500 ewes	> 500 ewes	≤ 165 ewes	166-330 ewes	331-500 ewes	> 500 ewes	
45	26	9	10	11	11	4	3	0.67
**Collaboration with a veterinarian**								
Yes	No			Yes	No			
73	17			28	1			0.044
**Age of the farmer**								
Up to 50 years	Over 50 years			Up to 50 years	Over 50 years			
55	35			18	11			0.93
**Length of previous animal farming experience of the farmer**								
≤ 5 years	> 5 years			≤ 5 years	> 5 years			
19	71			5	24			0.65
**Education of the farmer**								
Primary education	Secondary or post-secondary education	Tertiary education		Primary education	Secondary education	Tertiary education		
16	68	6		4	21	4		0.46
**Professional involvement in farming**								
Full-time	Part-time			Full-time	Part-time			
80	10			25	4			0.70
**Daily period at the farm**								
≤ 8 hours	> 8 hours			≤ 8 hours	> 8 hours			
20	70			8	21			0.55
**Family tradition in farming**								
Yes	No			Yes	No			
77	13			26	3			0.57
**Presence of working staff in the herd**								
Yes	No			Yes	No			
23	67			11	18			0.20

Table S14. Incidence rate of lamb / kid pneumonia or diarrhoea on 325 sheep flocks and 119 goat herds in Greece, in accordance with the routine administration of antibiotics to newborns.

Routine administration of antibiotics to newborns	Sheep flocks		Goat herds	
	Incidence rate of pneumonia	Incidence rate of diarrhoea	Incidence rate of pneumonia	Incidence rate of diarrhoea
Yes	2.2%	9.9%	2.4%	11.2%
No	1.5%	9.6%	1.1%	14.7%
p	< 0.0001	0.09	< 0.0001	< 0.0001

[1] number of sheep / goat farms.

Table S15. Results of the association of the incidence rate of lamb / kid pneumonia with the administration of antibiotics in those cases on 325 sheep flocks and 119 goat herds in Greece.

**Incidence rate**	**Sheep flocks**		**Goat herds**	
	Administration of antibiotics in cases of pneumonia	No administration of antibiotics in cases of pneumonia	Administration of antibiotics in cases of pneumonia	No administration of antibiotics in cases of pneumonia
≤ 3%	33 [1]	9	4	2
> 3%	38	13	14	3
p	0.21		0.42	

[1] number of sheep / goat farms.

Table S16. Results of univariable analysis for associations of farm-related factors with the use of antibiotics in cases of lamb pneumonia on 325 sheep flocks in Greece.

**Use of antibiotics (*n* = 71)**				**No use of antibiotics (*n* = 22)**				***p***
**Management system applied in the farm**								
Intensive	Semi-intensive	Semi-extensive	Extensive	Intensive	Semi-intensive	Semi-extensive	Extensive	
12	30	23	6	3	8	9	2	0.89
**No. of ewes in the flock**								
≤ 165 ewes	166-330 ewes	331-500 ewes	> 500 ewes	≤ 165 ewes	166-330 ewes	331-500 ewes	> 500 ewes	
17	25	16	13	4	9	7	2	0.60
**Breed of animals**								
Crossbreeds	Imported breeds	Local breeds		Crossbreeds	Imported breeds	Local breeds		
3	32	36		4	9	9		0.09
**Average age of culling ewes**								
≤ 6 years	> 6 years			≤ 6 years	> 6 years			
54	17			15	7			0.46
**Start of lambing period**								
All year	Autumn	Winter	Spring - Summer	All year	Autumn	Winter	Spring - Summer	
0	36	35	0	2	13	7	0	0.020
**Collaboration with a veterinarian**								
Yes	No			Yes	No			
61	10			18	4			0.64
**Method of calculation of bodyweight for the administration of antibiotics**								
Estimation	Weighing			Estimation	Weighing			
58	13			22	0			0.030
**Administration of antibiotics to animals at the dose prescribed**								
Administration at a higher dose than that prescribed	Administration at the dose prescribed			Administration at a higher dose than that prescribed	Administration at the dose prescribed			
17	54			3	19			0.30
**Induction of lambing**								
Yes	No			Yes	No			
3	68			0	22			0.33
**Newborn care and specific monitoring**								
Yes	No			Yes	No			
65	6			21	1			0.54

**Maintenance of a colostrum bank**						
Yes	No	Yes	No			
9	62	1	21			0.28
**Newborn fostering to female animals other than their dams**						
Yes	No	Yes	No			
47	24	15	7			0.86
**Vaccination against bacterial pneumonia**						
Yes	No	Yes	No			
21	50	7	15			0.84
**Administration of selenium to pregnant ewes**						
Yes	No	Yes	No			
22	49	5	17			0.46
**Administration of selenium to newborn lambs**						
Yes	No	Yes	No			
54	17	16	6			0.75
**Disinfection of the navel stump in newborns**						
Yes	No	Yes	No			
47	24	15	7			0.86
**Tail docking in newborns**						
Yes	No	Yes	No			
59	12	19	3			0.72
**Routine administration of antibiotics to newborns**						
Yes	No	Yes	No			
27	44	5	17			0.19
**Age of the farmer**						
Up to 50 years	Over 50 years	Up to 50 years	Over 50 years			
48	23	12	10			0.26
**Length of previous animal farming experience of the farmer**						
≤ 5 years	> 5 years	≤ 5 years	> 5 years			
16	55	6	16			0.65
**Education of the farmer**						
Primary education	Secondary or post-secondary education	Tertiary education	Primary education	Secondary education	Tertiary education	
16	49	9	5	9	8	0.034

**Professional involvement in farming**				
Full-time	Part-time	Full-time	Part-time	
68	3	21	1	0.95
**Daily period at the farm**				
≤ 8 hours	> 8 hours	≤ 8 hours	> 8 hours	
22	49	3	19	0.11
**Family tradition in farming**				
Yes	No	Yes	No	
59	12	22	0	0.039
**Presence of working staff in the flock**				
Yes	No	Yes	No	
31	40	12	10	0.37

Table S17. Results of univariable analysis for associations of farm-related factors with the use of antibiotics in cases of kid pneumonia on 119 goat herds in Greece.

Use of antibiotics (*n* = 17)				No use of antibiotics (*n* = 6)				*p*
**Management system applied in the farm**								
Intensive	Semi-intensive	Semi-extensive	Extensive	Intensive	Semi-intensive	Semi-extensive	Extensive	
1	6	8	2	1	0	3	2	0.27
**No. of does in the herd**								
≤ 165 ewes	166-330 ewes	331-500 ewes	> 500 ewes	≤ 165 ewes	166-330 ewes	331-500 ewes	> 500 ewes	
4	5	5	3	2	2	1	1	0.93
**Breed of animals**								
Crossbreeds	Imported breeds	Local breeds		Crossbreeds	Imported breeds	Local breeds		
0	9	8		1	1	4		0.11
**Average age of culling does**								
≤ 6 years	> 6 years			≤ 6 years	> 6 years			
9	8			4	2			0.56
**Start of kidding period**								
All year	Autumn	Winter	Spring - Summer	All year	Autumn	Winter	Spring - Summer	
1	6	10	0	0	3	3	0	0.72
**Collaboration with a veterinarian**								
Yes	No			Yes	No			
15	2			4	2			0.23
**Method of calculation of bodyweight for the administration of antibiotics**								
Estimation	Weighing			Estimation	Weighing			
13	4			6	0			0.19
**Administration of antibiotics to animals at the dose prescribed**								
Administration at a higher dose than that prescribed	Administration at the dose prescribed			Administration at a higher dose than that prescribed	Administration at the dose prescribed			
3	14			1	5			0.96
**Induction of lambing**								
Yes	No			Yes	No			
0	17			0	6			n/a
**Newborn care and specific monitoring**								
Yes	No			Yes	No			
12	5			4	2			0.86

**Maintenance of a colostrum bank**				
Yes	No	Yes	No	
2	15	1	5	0.76
**Newborn fostering to female animals other than their dams**				
Yes	No	Yes	No	
16	1	6	0	0.54
**Vaccination against bacterial pneumonia**				
Yes	No	Yes	No	
5	12	2	4	0.86
**Administration of selenium to pregnant does**				
Yes	No	Yes	No	
5	12	2	4	0.86
**Administration of selenium to newborn kids**				
Yes	No	Yes	No	
10	7	2	4	0.28
**Disinfection of the navel stump in newborns**				
Yes	No	Yes	No	
11	6	5	1	0.39
**Tail docking in newborns**				
Yes	No	Yes	No	
8	9	2	4	0.56
**Routine administration of antibiotics to newborns**				
Yes	No	Yes	No	
8	9	3	3	0.90
**Age of the farmer**				
Up to 50 years	Over 50 years	Up to 50 years	Over 50 years	
14	3	3	3	0.12
**Length of previous animal farming experience of the farmer**				
≤ 5 years	> 5 years	≤ 5 years	> 5 years	
4	13	1	5	0.73

**Education of the farmer**						
Primary education	Secondary or post-secondary education	Tertiary education	Primary education	Secondary education	Tertiary education	
5	9	3	1	5	0	0.37

**Professional involvement in farming**				
Full-time	Part-time	Full-time	Part-time	
14	3	5	1	0.96
**Daily period at the farm**				
≤ 8 hours	> 8 hours	≤ 8 hours	> 8 hours	
4	13	0	6	0.19
**Family tradition in farming**				
Yes	No	Yes	No	
15	2	6	0	0.38
**Presence of working staff in the flock**				
Yes	No	Yes	No	
4	13	2	4	0.64

Table S18. Results of the association of the incidence rate of lamb / kid diarrhoea with the administration of antibiotics in those cases on 325 sheep flocks and 119 goat herds in Greece.

**Incidence rate**	**Sheep flocks**		**Goat herds**	
	Administration of antibiotics in cases of diarrhoea	No administration of antibiotics in cases of diarrhoea	Administration of antibiotics in cases of diarrhoea	No administration of antibiotics in cases of diarrhoea
≤ 5%	49	13	8	3
> 5%	116	23	49	9
p	0.45		0.35	

[1] number of sheep / goat farms.

Table S19. Results of univariable analysis for associations of farm-related factors with the use of antibiotics in cases of lamb diarrhoea on 325 sheep flocks in Greece.

**Use of antibiotics (*n* = 158)**				**No use of antibiotics (*n* = 36)**				***p***
**Management system applied in the farm**								
Intensive	Semi-intensive	Semi-extensive	Extensive	Intensive	Semi-intensive	Semi-extensive	Extensive	
24	67	57	10	4	20	11	1	0.50
**No. of ewes in the flock**								
≤ 165 ewes	166-330 ewes	331-500 ewes	> 500 ewes	≤ 165 ewes	166-330 ewes	331-500 ewes	> 500 ewes	
43	56	33	26	9	12	9	6	0.96
**Breed of animals**								
Crossbreeds	Imported breeds	Local breeds		Crossbreeds	Imported breeds	Local breeds		
23	66	69		3	16	17		0.61
**Average age of culling ewes**								
≤ 6 years	> 6 years			≤ 6 years	> 6 years			
112	46			26	10			0.87
**Start of lambing period**								
All year	Autumn	Winter	Spring - Summer	All year	Autumn	Winter	Spring - Summer	
8	112	35	3	5	25	6	0	0.21
**Collaboration with a veterinarian**								
Yes	No			Yes	No			
141	17			27	9			0.023
**Method of calculation of bodyweight for the administration of antibiotics**								
Estimation	Weighing			Estimation	Weighing			
136	22			27	9			0.10
**Administration of antibiotics to animals at the dose prescribed**								
Administration at a higher dose than that prescribed	Administration at the dose prescribed			Administration at a higher dose than that prescribed	Administration at the dose prescribed			
24	134			11	25			0.030
**Induction of lambing**								
Yes	No			Yes	No			
2	156			0	36			0.50
**Newborn care and specific monitoring**								
Yes	No			Yes	No			
144	14			32	4			0.67

**Maintenance of a colostrum bank**				
Yes	No	Yes	No	
21	137	5	31	0.92
**Newborn fostering to female animals other than their dams**				
Yes	No	Yes	No	
108	50	22	14	0.40
**Vaccination against clostridial infections**				
Yes	No	Yes	No	
154	4	35	1	0.93
**Administration of selenium to pregnant ewes**				
Yes	No	Yes	No	
48	110	10	26	0.76
**Administration of selenium to newborn lambs**				
Yes	No	Yes	No	
118	40	25	11	0.52
**Disinfection of the navel stump in newborns**				
Yes	No	Yes	No	
101	57	22	14	0.75
**Tail docking in newborns**				
Yes	No	Yes	No	
122	36	25	11	0.33
**Routine administration of antibiotics to newborns**				
Yes	No	Yes	No	
44	114	4	32	0.036
**Age of the farmer**				
Up to 50 years	Over 50 years	Up to 50 years	Over 50 years	
101	57	25	11	0.53
**Length of previous animal farming experience of the farmer**				
≤ 5 years	> 5 years	≤ 5 years	> 5 years	
36	122	9	127	0.78

**Education of the farmer**						
Primary education	Secondary or post-secondary education	Tertiary education	Primary education	Secondary education	Tertiary education	
31	108	19	7	25	4	0.99

**Professional involvement in farming**				
Full-time	Part-time	Full-time	Part-time	
145	13	32	4	0.58
**Daily period at the farm**				
≤ 8 hours	> 8 hours	≤ 8 hours	> 8 hours	
41	117	13	23	0.22
**Family tradition in farming**				
Yes	No	Yes	No	
137	21	30	6	0.60
**Presence of working staff in the flock**				
Yes	No	Yes	No	
64	94	10	26	0.16

Table S20. Results of univariable analysis for associations of farm-related factors with the use of antibiotics in cases of kid diarrhoea on 119 goat herds in Greece.

**Use of antibiotics (*n* = 57)**				**No use of antibiotics (*n* = 12)**				***p***
**Management system applied in the farm**								
Intensive	Semi-intensive	Semi-extensive	Extensive	Intensive	Semi-intensive	Semi-extensive	Extensive	
3	17	29	8	1	4	4	3	0.66
**No. of does in the herd**								
≤ 165 ewes	166-330 ewes	331-500 ewes	> 500 ewes	≤ 165 ewes	166-330 ewes	331-500 ewes	> 500 ewes	
22	19	8	8	3	5	1	3	0.64
**Breed of animals**								
Crossbreeds	Imported breeds	Local breeds		Crossbreeds	Imported breeds	Local breeds		
8	26	23		1	2	9		0.09
**Average age of culling does**								
≤ 6 years	> 6 years			≤ 6 years	> 6 years			
22	35			4	8			0.73
**Start of kidding period**								
All year	Autumn	Winter	Spring - Summer	All year	Autumn	Winter	Spring - Summer	
2	29	26	0	0	8	3	1	0.08
**Collaboration with a veterinarian**								
Yes	No			Yes	No			
46	11			10	2			0.83
**Method of calculation of bodyweight for the administration of antibiotics**								
Estimation	Weighing			Estimation	Weighing			
44	13			8	4			0.44
**Administration of antibiotics to animals at the dose prescribed**								
Administration at a higher dose than that prescribed	Administration at the dose prescribed			Administration at a higher dose than that prescribed	Administration at the dose prescribed			
12	45			4	8			0.36
**Induction of lambing**								
Yes	No			Yes	No			
1	56			0	12			0.64
**Newborn care and specific monitoring**								
Yes	No			Yes	No			
50	7			12	0			0.20

**Maintenance of a colostrum bank**						
Yes	No	Yes	No			
4	53	2	10			0.28
**Newborn fostering to female animals other than their dams**						
Yes	No	Yes	No			
15	42	2	10			0.48
**Vaccination against clostridial infections**						
Yes	No	Yes	No			
30	27	4	8			0.22
**Administration of selenium to pregnant does**						
Yes	No	Yes	No			
15	42	2	10			0.48
**Administration of selenium to newborn kids**						
Yes	No	Yes	No			
30	27	4	8			0.22
**Disinfection of the navel stump in newborns**						
Yes	No	Yes	No			
30	27	5	7			0.49
**Tail docking in newborns**						
Yes	No	Yes	No			
19	38	4	8			0.99
**Routine administration of antibiotics to newborns**						
Yes	No	Yes	No			
19	38	1	11			0.08
**Age of the farmer**						
Up to 50 years	Over 50 years	Up to 50 years	Over 50 years			
34	23	9	3			0.32
**Length of previous animal farming experience of the farmer**						
≤ 5 years	> 5 years	≤ 5 years	> 5 years			
7	50	2	10			0.68
**Education of the farmer**						
Primary education	Secondary or post-secondary education	Tertiary education	Primary education	Secondary education	Tertiary education	
11	40	6	3	9	0	0.48

**Professional involvement in farming**				
Full-time	Part-time	Full-time	Part-time	
50	7	12	0	0.20
**Daily period at the farm**				
≤ 8 hours	> 8 hours	≤ 8 hours	> 8 hours	
14	43	1	11	0.22
**Family tradition in farming**				
Yes	No	Yes	No	
52	5	11	1	0.96
**Presence of working staff in the flock**				
Yes	No	Yes	No	
17	40	1	11	0.12
